# E3 ubiquitin ligase MARCH5 positively regulates Japanese encephalitis virus infection by catalyzing the K27-linked polyubiquitination of viral E protein and inhibiting MAVS-mediated type I interferon production

**DOI:** 10.1128/mbio.00208-25

**Published:** 2025-03-12

**Authors:** Chenxi Li, Chenyang Tang, Xiqian Liu, Ying Liu, Linjie Zhang, Jing Shi, Qingyu Li, Mingan Sun, Yanhua Li

**Affiliations:** 1 College of Veterinary Medicine, Yangzhou University, Yangzhou, Jiangsu, China; 2 Comparative Medicine Research Institute, Yangzhou University, Yangzhou, Jiangsu, China; 3 Jiangsu Co-innovation Center for Prevention and Control of Important Animal Infectious Diseases and Zoonoses, Yangzhou, Jiangsu, China; 4 Shanghai Key Laboratory of Veterinary Biotechnology, School of Agriculture and Biology, Shanghai Jiao Tong University, Shanghai, China; 5 College of Life Science, Anqing Normal University, Anqing, Anhui, China; Huazhong Agricultural University, Wuhan, Hubei, China

**Keywords:** Japanese encephalitis virus, MARCH5, ubiquitination, MAVS, RLR signaling pathway

## Abstract

Membrane-associated RING-CH-type finger (MARCH) proteins, a class of E3 ubiquitin ligases, have been reported to be involved in the infection of multiple viruses and the regulation of type I interferon (IFN) production. However, the specific role and mechanisms by which MARCH proteins influence Japanese encephalitis virus (JEV) infection remain poorly understood. Here, we systematically investigate the functional relevance of MARCH proteins in JEV replication by examining the effects of siRNA-mediated knockdown of MARCHs on viral infection. We identified MARCH5 as a positive regulator of JEV replication. The knockout of MARCH5 dramatically reduced viral yields, whereas its overexpression significantly enhanced JEV replication. Mechanistically, MARCH5 specifically interacts with the JEV envelope (E) protein and promotes its K27-linked polyubiquitination at the lysine (K) residues 136 and 166. This ubiquitination enhances viral attachment to permissive cells. Substituting these lysine residues with arginine (R) attenuated JEV replication *in vitro* and reduced viral virulence *in vivo*. Furthermore, JEV infection upregulated the expression of MARCH5. We also discovered that MARCH5 degrades mitochondrial antiviral-signaling protein (MAVS) through the ubiquitin-proteasome pathway by catalyzing its K48-linked ubiquitination, thereby inhibiting type I IFN production in JEV-infected cells. This suppression of type I IFN further facilitates JEV infection. In conclusion, these findings disclosed a novel role of MARCH5 in positively regulating JEV infection and revealed an important mechanism employed by MARCH5 to regulate the innate immune response.

**IMPORTANCE:**

JEV is the leading cause of viral encephalitis in many countries of Asia with an estimated 100,000 clinical human cases and causes economic loss to the swine industry. Until now, there is no clinically approved antiviral for the treatment of JEV infection. Although vaccination prophylaxis is widely regarded as the most effective strategy for preventing Japanese encephalitis (JE), the incidence of JE cases continues to rise. Thus, a deeper understanding of virus-host interaction will enrich our knowledge of the mechanisms underlying JEV infection and identify novel targets for the development of next-generation live-attenuated vaccines and antiviral therapies. To the best of our knowledge, this study is the first to identify MARCH5 as a pro-viral host factor that facilitates JEV infection. We elucidated two distinct mechanisms by which MARCH5 promotes JEV infection. First, MARCH5 interacts with viral E protein and mediates the K27-linked ubiquitination of E protein at the K136 and K166 residues to facilitate efficient viral attachment. Furthermore, double mutations of K136R-K166R attenuated JEV infection *in vitro* and reduced viral virulence in mice. Second, the upregulated expression of MARCH5 induced by JEV infection further suppresses the RIG-I-like receptor (RLR) signaling pathway to benefit viral infection. MARCH5 downregulates type I IFN production by conjugating the K48-linked polyubiquitin at the K286 of MAVS, which leads to MAVS degradation through the ubiquitin-proteasome pathway. In summary, this study provides novel insights into the role played by MARCH proteins in JEV infection and identifies specific ubiquitination sites on JEV E protein that could be targeted for viral attenuation and the development of antiviral therapeutics.

## INTRODUCTION

Japanese encephalitis virus (JEV) is an important arthropod-borne zoonotic pathogen that can cause severe viral encephalitis in humans (1). Since its first outbreak in Japan in 1924, JEV has become endemic in most countries in Asia and Oceania. According to the World Health Organization (WHO), over three billion people in 24 countries are at risk of JEV infection (2). The global incidence of JE is estimated at approximately 68,000 cases annually, with a fatality rate of 10%–15% and up to 50% of survivors experiencing permanent neurological or psychiatric sequelae (3, 4). Currently, JEV is the leading cause of viral encephalitis in Asia (1). As an arthropod-borne virus, JEV transmits between mosquito vectors and vertebrate amplifying hosts (pigs and wading birds) in nature (5, 6). As the amplifying hosts, pigs play a crucial role in JEV transmission due to their ability to develop sufficiently high viremia to infect mosquitoes (7). Although JEV infection in commercial pigs is generally asymptomatic, it can cause reproductive diseases such as abortion and stillbirth in pregnant sows (6). Therefore, effective prevention and control of JEV transmission in pigs are crucial for both the swine industry and public health.

JEV is a member of the genus *Orthoflavivirus* in the *Flaviviridae* family, which includes many important zoonotic pathogens, such as Zika virus (ZIKV), Dengue virus (DENV), and Tick-borne encephalitis virus (TBEV) (8, 9). Like other flaviviruses, JEV is an enveloped virus containing an RNA genome of approximately 11 kb encapsulated by capsid proteins (C) and surrounded by an icosahedral shell consisting of the envelope glycoprotein (E) and the membrane or precursor membrane protein (M/prM) anchored in a lipid membrane (9, 10). On the surface of the mature virion, the flavivirus E is an antiparallel dimer with a fusion loop that consists of three ectodomains (EDI, EDII, and EDIII), a stem region, and transmembrane (TM) regions (9, 11). As the only mature glycosylated protein, E protein is profoundly involved in viral attachment, membrane fusion, and cell entry (10), contributing significantly to JEV virulence and host-specific adaptation. Comparative genomic analysis of JEV strains with varying virulence has identified specific amino acid (aa) variations in the E protein associated with JEV neurovirulence or neuroinvasiveness. For instance, the G306E mutation in the E protein reduced the binding affinity between the virion and cellular receptors, diminishing neurotoxicity (12). The acidity/alkalinity of the 138^th^ residue affects virion binding to neuronal cells, thereby influencing JEV neurovirulence (13). Additionally, residues at positions 107 (14), 279 (15), and 389 (10) have been identified as the key determinants of JEV neurovirulence, although the underlying mechanisms remain unclear.

Ubiquitination is a post-translational modification process mediated by a cascade of enzymes, including E1 ubiquitin-activating enzymes, E2 ubiquitin-conjugating enzymes, and E3 ubiquitin ligases (16, 17). In contrast to the limited number of E1 and E2 enzymes, several hundred E3 ubiquitin ligases confer substrate specificity to ubiquitination. Membrane-associated RING-CH-type finger (MARCH) proteins are a class of E3 ubiquitin ligases (18). In mammals, MARCH family consists of 11 members, including MARCH1 to 11 (19), each harboring a catalytic domain with a highly conserved C4HC3 cysteine-histidine (RING-CH finger) configuration in their N-terminal cytoplasmic tail and transmembrane domains (20). MARCH proteins are widely distributed and regulate diverse cellular functions, including antiviral innate immune signaling pathways, transmembrane transport of proteins, and endosomal protein trafficking (19, 21). Protein ubiquitination plays an important role in innate immune responses by modulating components of RLR-mediated signaling, such as RIG-I, MAVS, TRAF3, TBK1, and IRF3 (16, 22). Previous studies have demonstrated that MARCH proteins regulated the RLR signaling pathway through their E3 ubiquitin ligase activity. For example, MARCH8 targets MAVS through K29-linked polyubiquitination to inhibit RLR signaling (23), whereas MARCH7 interacts with ubiquitinated TBK1 to suppress type I IFN production (24). MARCH1 inhibits STING-induced type I IFN production by catalyzing the K6-, K11-, K27-, and K29-linked ubiquitination of STING (25). Thus, different members of the MARCH family may regulate type I IFN production through distinct mechanisms.

As obligate intracellular parasites, viruses often hijack the ubiquitination processes to modify their proteins, facilitating various stages of their life cycle. In flaviviruses, the ubiquitination of viral proteins plays diverse roles in viral replication and pathogenesis. For example, the ubiquitination of ZIKV envelope protein promotes its interaction with cellular receptors, which has a broad impact on virus entry, host tropism, and pathogenesis (26). The ubiquitination of the DENV C protein is required for efficient viral genome release (27), whereas the K27-linked ubiquitination of the TBEV NS4A protein at lys132 is essential for its antagonism of type I IFN-dependent signaling (28). Although ubiquitination of several JEV proteins, such as NS3 and NS5, has been reported (29), the functional significance of ubiquitinated viral proteins in JEV infection is largely unclear. In this study, we identified the E3 ubiquitin ligase MARCH5 as a pro-viral factor to JEV infection. We demonstrated that MARCH5 interacts with JEV E protein to promote its K27-linked polyubiquitination at K136 and K166 residues, which enhance viral attachment. Besides, MARCH5 catalyzes K48-linked ubiquitination of MAVS at K286, which triggers the degradation of MAVS through the ubiquitin-proteasome pathway, resulting in the inhibition of type I IFN response. These findings reveal a novel role for MARCH5 in promoting JEV infection and elucidate a key mechanism by which MARCH5 downregulates the RLR signaling pathway to facilitate viral replication.

## RESULTS

### MARCH5 positively regulated JEV replication

**Fig 1:**
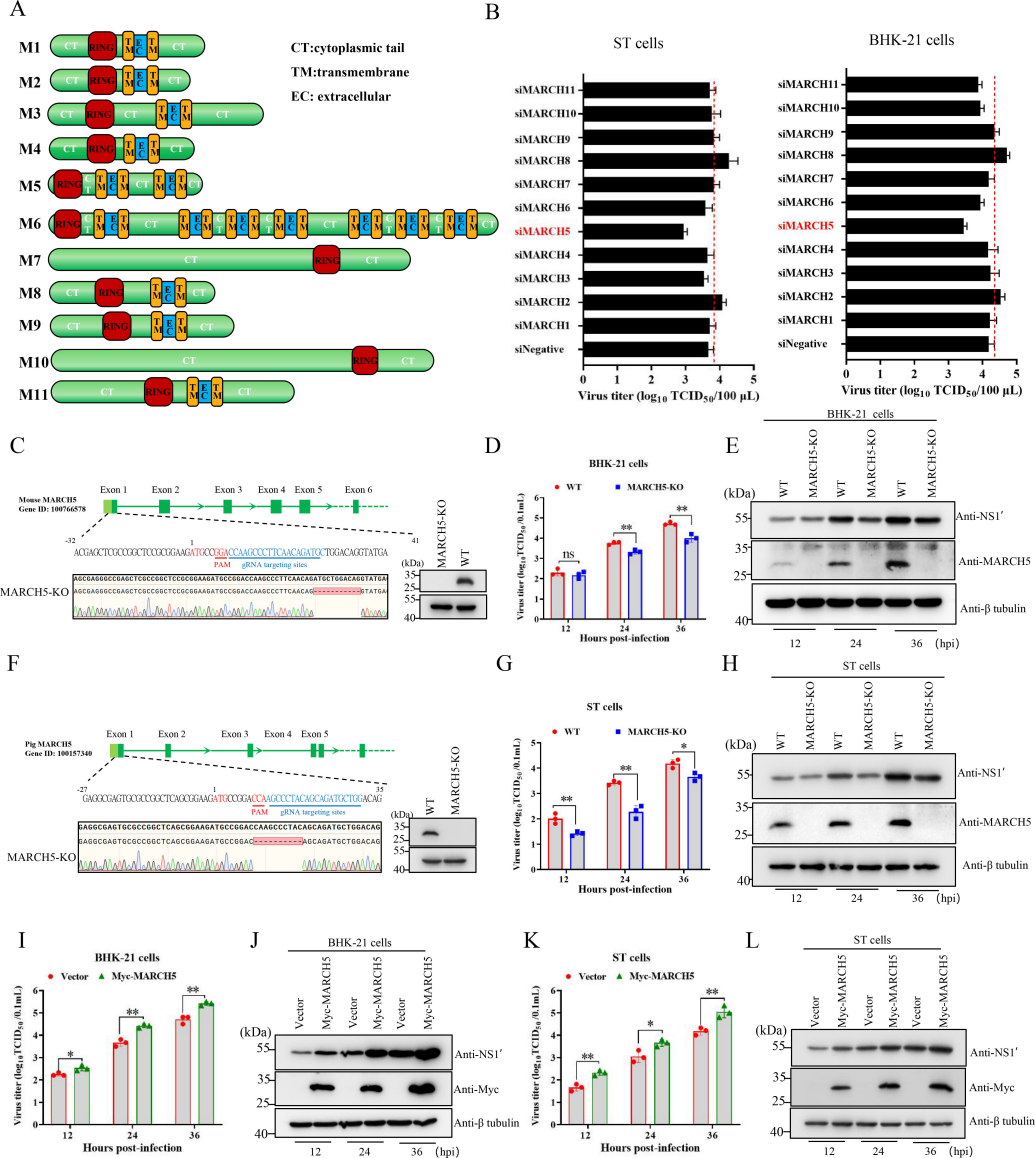
MARCH5 is involved in JEV replication. (**A**) Schematic representation of MARCH family members. CT, cytoplasmic tail; TM, transmembrane; EC, extracellular. (**B**) ST or BHK-21 cells were treated with siRNA (50 pmol) targeting MARCH protein expression (siRNA+) or scrambled RNA control (siNegative) for 12 h, followed by infection with JEV at an MOI of 0.1. At 24 hpi, viral titers in culture supernatants were measured using TCID_50_ assays. (**C and F**) Sequence analysis of MARCH5 in WT and MARCH5-KO BHK-21 (**C**) and ST (**F**) cell lines (left). MARCH5 expression in WT and MARCH5-KO BHK-21 (**C**) and ST (**F**) cell lines was validated by Western blotting (right). (**D, E, G, and H**) WT and MARCH5-KO ST and BHK-21 cells were infected with JEV at an MOI of 0.1. Viral titers in culture supernatants at indicated time points were determined by calculating the log_10_TCID_50_/0.1 mL (**D and G**), and the expression of NS1' and MARCH5 proteins was detected by immunoblotting (**E and H**). (**I–L**) BHK-21 or ST cells were transfected with a Myc-MARCH5 expression construct and infected with JEV at an MOI of 0.1. The viral titers at the indicated time points were measured by TCID_50_ assay (**I and K**), and the expression of NS1’ and Myc-MARCH5 proteins was detected by immunoblotting (**J and L**). Data are represented as means ± SD from three independent experiments. Statistical significance was determined using a two-tailed unpaired Student’s *t*-test: *, *P* < 0.05; **, *P* < 0.01; ***, *P* < 0.001; ns, no statistically significant.

MARCH family consists of 11 members, including MARCH1 to MARCH11. All members share a conserved N terminus RING-CH motif (RING) and typically multiple transmembrane domains (TM), except for MARCH7 and MARCH10, which lack predicted TM domains (Fig. 1A). Initially, a gene-silencing screen using siRNA targeting MARCH proteins (Table S1) in ST and BHK-21 lines was performed to identify the members that are involved in JEV replication. The efficacy of siRNA-mediated downregulation of MARCHs mRNA expression was confirmed by qRT-PCR in both BHK-21 and ST cells (Fig.S1A and B). Among the 11 MARCH proteins screened, MARCH5 was identified to positively regulate JEV replication. Knockdown of MARCH5 in ST and BHK-21 cells led to an approximately 10-fold reduction of virus yields at 24 h post-infection (hpi) (Fig. 1B). To further validate the pro-viral role of MARCH5, we generated MARCH5-knockout (KO) cell lines in both BHK-21 and ST cells using the CRISPR/cas9 gene editing technology. Deletions of 11-nucleotide and 10-nucleotide within the exon 1 of MARCH5 were identified in the MARCH5-KO cell lines of BHK-21 and ST cell lines, respectively (Fig. 1C and F). Western blot analysis confirmed the absence of MARCH5 protein in these KO cell lines (Fig. 1C and F). The MARCH5-KO cells exhibited growth kinetics similar to their parental cell lines, as determined by the CCK-8 assay (Fig. S1C and D). Subsequently, to evaluate the impact of MARCH5-KO on JEV replication, wild-type (WT) and MARCH5-KO cell lines were inoculated with JEV strain Beijing/2020–1 at an MOI of 0.1. Virus titers in the supernatant were measured at 12, 24, and 36 hpi. Compared with WT cells, MARCH-KO BHK-21 cells showed 2.91-fold and 3.02-fold decreases in viral titers at 24 and 36 hpi, respectively (Fig. 1D). Similarly, MARCH5-KO ST cells exhibited 3.95-fold, 14.66-fold, and 3.8-fold reductions in viral titers at 12, 24, and 36 hpi (Fig. 1G). Consistent with these findings, the levels of viral NS1' protein were notably lower in MARCH5-KO cells compared with WT cells (Fig. 1E and H). These results collectively demonstrate that MARCH5 is directly involved in JEV replication.

We next investigated the response of MARCH5 to JEV infection. As shown in Fig. S2, the mRNA and protein expression levels of endogenous MARCH5 were dramatically upregulated following JEV infection (Fig.S2A through D). Given this induction, we evaluated the effect of MARCH5 overexpression on JEV replication. BHK-21 and ST cells were respectively transfected with a Myc-MARCH5 expression construct and then infected with JEV at an MOI of 0.1. Overexpression of MARCH5 resulted in 1.92-fold to 5.75-fold and 4.11-fold to 7.36-fold increases in viral yields in BHK-21 and ST cells, respectively (Fig. 1I and K). The expression levels of viral NS1' were also obviously increased in MARCH5-overexpressing cells compared with the control cells (Fig. 1J and L).

Together, both loss-of-function and gain-of-function studies demonstrate that E3 ubiquitin ligase MARCH5 positively regulates JEV replication.

### MARCH5 catalyzes K27-Linked polyubiquitination of the lysine residues at positions 136 and 166 of the JEV E protein

**Fig 2:**
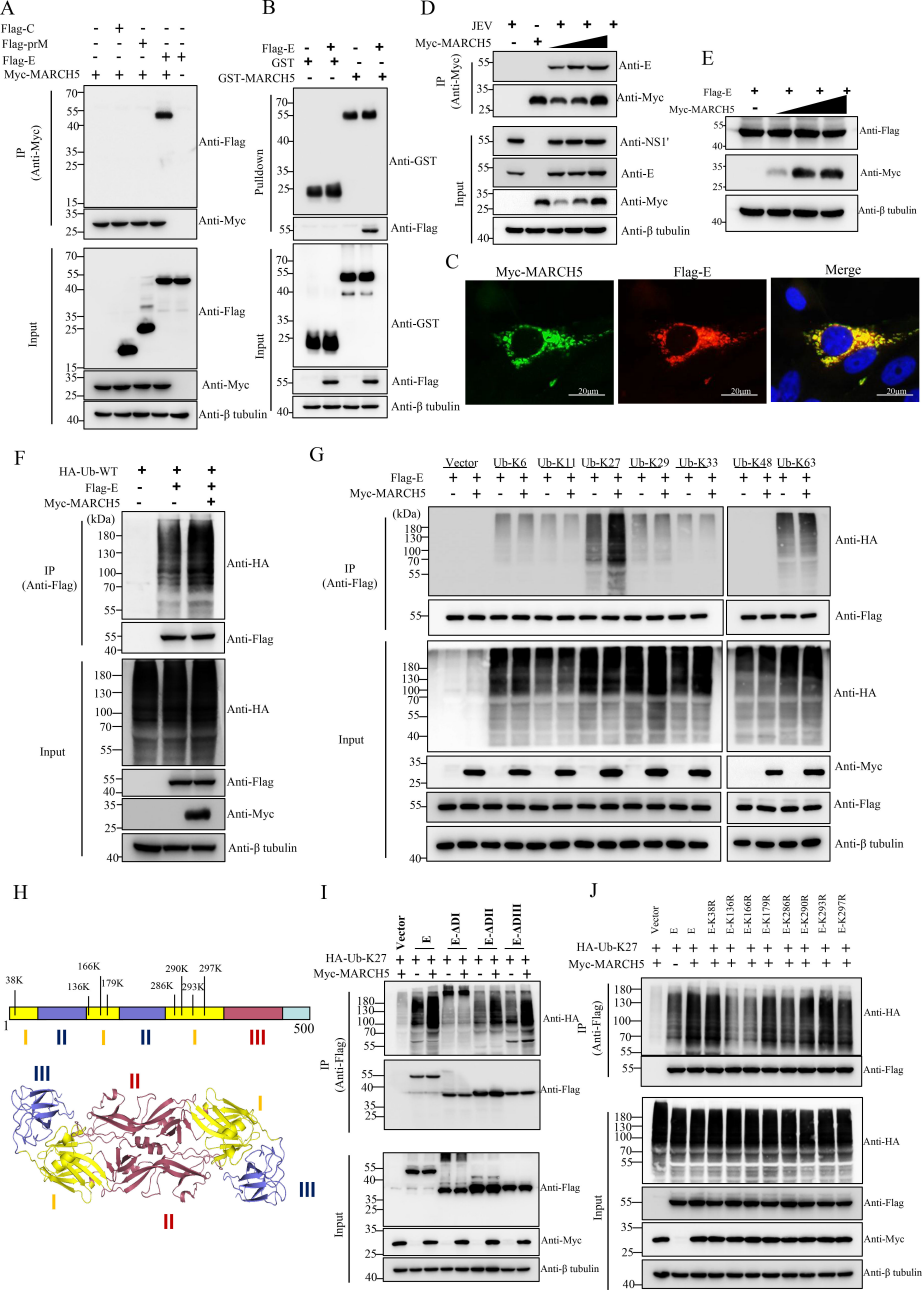
MARCH5 catalyzes K27-Linked polyubiquitination of lysine residues within the E protein. (**A**) HEK-293T cells were respectively transfected with plasmids expressing Flag-C, Flag-prM, or Flag-E, along with pMyc-MARCH5. After 24 h, the cells were subjected to co-IP assays with anti-Myc beads, followed by immunoblotting with anti-Myc and HA antibodies. (**B**) GST pulldown analysis of the interaction between recombinant GST-MARCH5 and Flag-E. GST and GST-MARCH5 expressed in *E. coli* BL21 were purified with glutathione beads and incubated with Flag-E. Bound proteins were analyzed by immunoblotting with anti-GST and anti-Flag antibodies. (**C**) BHK-21 cells were co-transfected with plasmids expressing Flag-E and Myc-MARCH5 for 24 h. Cells were fixed, permeabilized, and blocked, followed by incubation with anti-Myc (green) and anti-Flag (red) antibodies. Nuclei were stained with DAPI (blue). Scale bar: 20 µm. (**D**) BHK-21 cells were transfected with increasing amounts of plasmids expressing Myc-MARCH5 (250, 500, and 1,000 ng) for 12 h, followed by JEV infection at an MOI of 0.1 for 24 h. Cells were subjected to co-IP assays with anti-Myc beads and immunoblotted with anti-Myc and E antibodies. (**E**) BHK-21 were transfected with increasing amounts of plasmids expressing Myc-MARCH5 (250, 500, and 1,000 ng), along with pFlag-E. After 24 h, the cells were harvested to detect the expression of Myc-MARCH5 and Flag-E proteins. (**F and G**) Co-IP and immunoblot analysis of E protein ubiquitination in HEK-293T cells overexpressing MARCH5-Flag, along with HA-Ub-WT or HA-Ub mutants (Ub-K6, Ub-K11, Ub-K27, Ub-K29, Ub-K33, Ub-K48, and Ub-K63). (**H**) 3D structure of JEV E protein. EDI (yellow), EDII (blue), and EDIII (red) are highlighted. (**I and J**) HEK293T cells were co-transfected with pFlag-E or its mutants, pHA-Ub-K27, and pMyc-MARCH5 for 24 h. Lysates were collected for immunoprecipitation assays using anti-Flag magnetic beads. The ubiquitination of the E proteins was analyzed by immunoblotting with an anti-HA antibody.

MARCH proteins participate in the regulation of viral infection by targeting viral envelope glycoproteins (30, 31). Several MARCH proteins have recently been reported to target retroviral envelope glycoproteins (Env) and vesicular stomatitis virus G glycoprotein (VSV-G) (31–33). We therefore investigated the potential interaction between MARCH5 and JEV structural proteins using an overexpression system. Co-immunoprecipitation (co-IP) experiments suggested that MARCH5 interacts with JEV E protein but not with other structural proteins of JEV (Fig. 2A). The colocalization between MARCH5 and E protein in the cytoplasm was confirmed by confocal microscopy (Fig. 2C). Additionally, GST pulldown assays showed that GST-MARCH5, but not GST, pulldown Flag-tagged E, suggesting that a direct interaction exists between MARCH5 and the E protein (Fig. 2B). We also validated this interaction in JEV-infected BHK-21 cells, where increasing levels of MARCH5 expression correlated with enhanced immunoprecipitation of the viral E protein (Fig. 2D).

In flaviviruses, ubiquitination of the E protein plays an important role in virus replication (26). Since MARCH proteins often mediate the degradation of target proteins through their ubiquitin E3 ligase activity (23, 33), we first explored whether MARCH5 could degrade JEV E protein. The increasing amounts of MARCH5 did not affect the expression of the E protein (Fig. 2E) but enhanced the ubiquitination of E, as determined by IP and ubiquitination assays (Fig. 2F). This suggests that MARCH5 plays a role in the polyubiquitination of the JEV E protein rather than its degradation. The specific type of polyubiquitination chains linked to E protein catalyzed by MARCH5 was determined by using plasmids expressing seven ubiquitin mutants containing single lysine residue (K6, K11, K27, K29, K33, K48, or K63). Ubiquitination assays revealed that the E protein was associated with multiple types of polyubiquitination chains, including K27, K29, and K63. However, MARCH5 specifically enhanced the K27-linked ubiquitination of the E protein (Fig. 2G), indicating that MARCH5 mediates the K27-linked polyubiquitination of the JEV E protein.

Protein ubiquitination is a process of virtually transferring ubiquitin molecules to lysine residues on target proteins (16). JEV E protein consists of three structural domains (EDI, EDII, and EDIII) and contains multiple lysine residues distributed across these domains (Fig. 2H). To map the domain(s) of the E protein ubiquitinated by MARCH5, we generated three E mutants with individual domain deletion (E-ΔDI, E-ΔDII, and E-ΔDIII). Ubiquitination assays indicated that K27-linked ubiquitination was increased for the full-length E protein, E-ΔDII, and E-ΔDIII in the presence of MARCH5, but not for E-ΔDI (Fig. 2I), suggesting that EDI contains the ubiquitination sites recognized by MARCH5. Using the UbPred program (http://www.ubpred.org/), eight potential ubiquitination sites (Lys38, Lys136, Lys166, Lys179, Lys286, Lys290, Lys293, and Lys297) within the EDI domain were predicted (Fig. 2H). We then created a panel of E mutants (E-K38R, K136R, K166R, K179R, K286R, K290R, K293R, or K297R) carrying single lysine-to-arginine substitution at these sites. Ubiquitination assays demonstrated that MARCH5 increased K27-linked polyubiquitination of the WT E protein and all mutants except E-K136R and E-K166R mutants (Fig. 2J). These results indicate that MARCH5 specifically catalyzes K27-linked polyubiquitination of lysine residues at positions 136 and 166 of the JEV E protein.

### Lys136 and Lys 166 of the JEV E protein are determinants of viral replication and virulence

**Fig 3:**
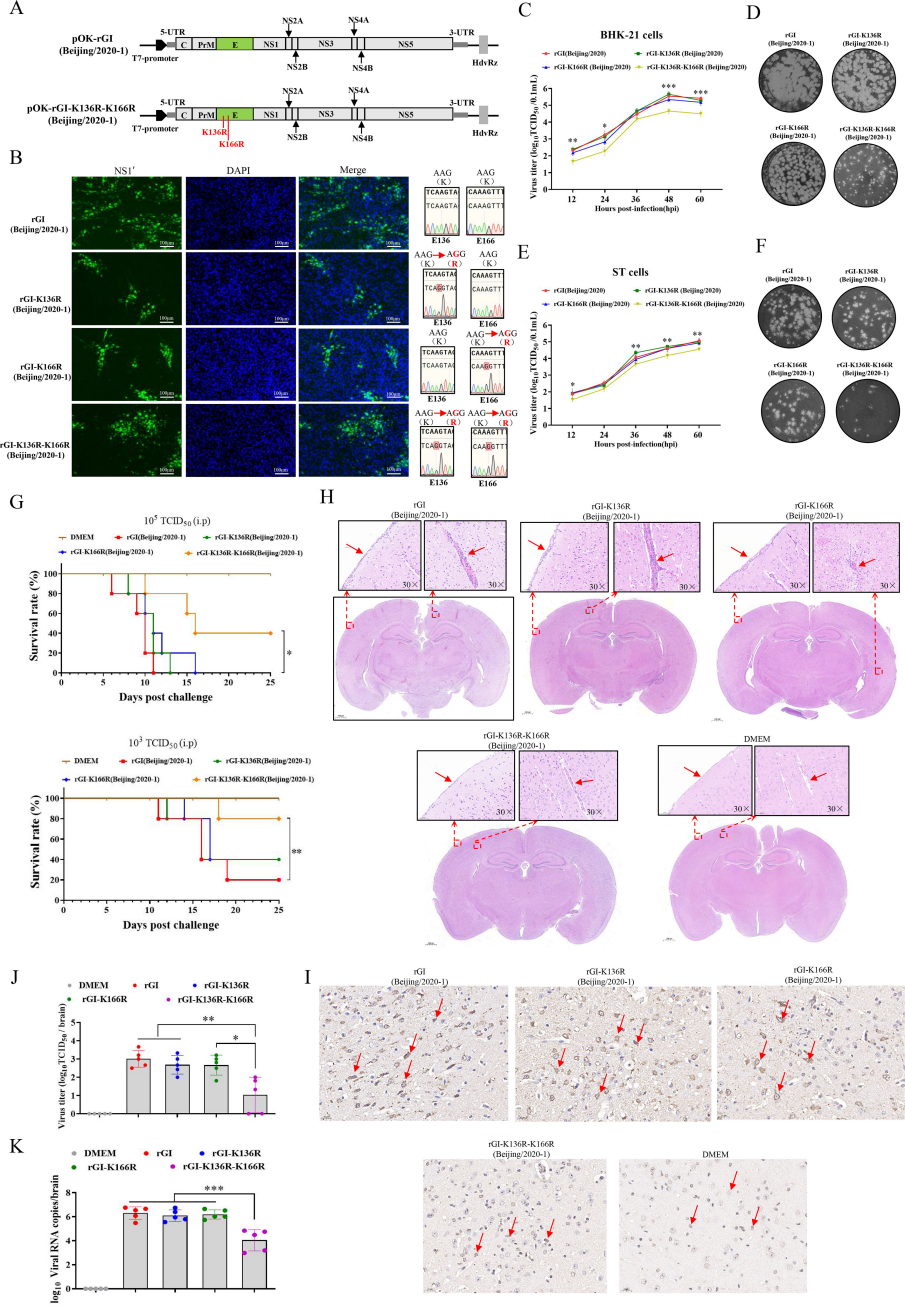
Lys136 and Lys166 of the E protein co-determine JEV replication and virulence. (A) Schematic representation of the strategy for rescuing mutant viruses. (B) BHK-21 cells were infected with fifth-passage viruses rGI, rGI-K136R, rGI-K166R, and rGI-K136R-K166R at an MOI of 0.1 for 24 h. Cells were stained with anti-NS1 antibody by IFA. Scale bar: 100 μm. Mutations of K136R and K166R were confirmed by Sanger sequencing. (C and E) BHK-21 and ST cells were infected with rGI, rGI-K136R, rGI-K166R, and rGI-K136R-K166R at an MOI of 0.05. Viral titers in supernatants were determined by TCID_50_ assay. Significant differences between rGI and rGI-K136R-K166R are marked (***, *P* < 0.001; **, *P* < 0.01; *, *P* < 0.05). (D and F) BHK-21 or ST cells were infected with viruses at 500~50 TCID_50_ for plaque morphology analysis. Plaques were visualized by crystal violet staining at 4 dpi. (G) Survival curves of mice infected with rGI, rGI-K136R, rGI-K166R, and rGI-K136R-K166R. Mice (*n* = 5) were intraperitoneally (i.p.) infected with 10^3^ and 10^5^ TCID_50_ of the virus and monitored for 25 days. Survival rates were calculated and plotted (***, *P* < 0.001; **, *P* < 0.01; The significant differences determined by the Kaplan-Meier analysis. (H and I) Brain samples from infected mice exhibiting neurological symptoms of paresis and tremors were collected for hematoxylin-and-eosin staining (H) and immunohistochemical (IHC) staining (I). (J and K) Mice (*n* = 5) were i.p. challenged with 10^5^ TCID_50_ of rGI, rGI-K136R, rGI-K166R, or rGI-K136R-K166R. Brain samples were collected at 6 dpi for viral titer determined by TCID_50_ assay (J) and viral genome quantification by RT-qPCR (K). Data are presented as mean ± SD from three independent experiments. The *P*-value was calculated using the *t*-test. ***, *P* < 0.001; **, *P* < 0.01; *, *P* < 0.05.

To investigate the functional role of lys136 and lys166 residues of the E protein, we generated three JEV mutants (rGI-K136R, rGI-K166R, and rGI-K136R-K166R) using a reverse genetics system of JEV Beijing/2020–1 strain (34) (Fig. 3A). The successful rescue of these mutants was confirmed by IFA, which showed strong NS1' expression (Fig. 3B). Additionally, the genetic stability of rGI-K136R, rGI-K166R, and rGI-K136R-K166R was determined via serially passaged in BHK-21 cells for five generations, with no reversion mutations observed at the E-K136R and E-K166R sites, as confirmed by genomic sequencing (Fig. 3B). To examine the impact of these mutations on JEV replication, the growth kinetics of the viruses rGI-K136R, rGI-K166R, rGI-K136R-K166R, and WT rGI was determined in BHK-21 and ST cells inoculated at an MOI of 0.05. Although rGI-K136R and rGI-K166R exhibited growth kinetics similar to the WT rGI, the double mutant rGI-K136R-K166R showed significantly reduced viral yields, with a 1.67-fold to 9.48-fold and 2.19-fold to 3.26-fold decrease in BHK-21 and ST cells, respectively, from 12 hpi to 60 hpi (Fig. 3C and E). Consistently, rGI-K136R-K166R formed smaller plaques compared with the WT rGI and single mutants (Fig. 3D and F). These data demonstrate that simultaneous arginine substitutions at lys136 and lys166 attenuated the replication ability of JEV *in vitro*.

Flaviviruses E proteins have been implicated in neurotoxicity (13, 35). To test whether lys136 and lys166 of the E protein contribute to the neuroinvasiveness of JEV, we conducted pathogenicity studies in weanling mice. Eight groups of mice (*n* = 5 per group) were respectively, intraperitoneally (i.p.) inoculated with rGI-K136R, rGI-K166R, rGI-K136R-K166R, or the WT rGI at doses of 10^3^ and 10^5^ TCID_50_. Survival curves revealed that mice infected with the rGI, rGI-K136R, or rGI-K166R strain had mortality rates of 100% at doses of 10^5^ TCID_50_ and mortality rates of 80%, 60%, and 80%, respectively, at doses of 10^3^ TCID_50_ (Fig. 3G). In contrast, the mortality rate of mice infected with rGI-K136R-K166R was significantly lower, with 60% at a dose of 10^5^ TCID_50_ and 20% at 10^3^ TCID_50_ (Fig. 3G). Histopathology examination further supported these findings, showing more severe encephalitis characterized by the multifocal lymphohistiocytic perivascular cuffs and lymphohistiocytic meningitis in the brains of mice infected with the WT rGI or single mutants compared with those infected with rGI-K136R-K166R (Fig. 3H). Additionally, viral antigen expression was markedly higher in the brains of mice infected with the WT rGI or single mutants than in those infected with rGI-K136R-K166R (Fig. 3I). Mice infected with the rGI-K136R-K166R strain showed lower viral load in their brain tissue compared with those infected with the WT rGI or single mutants at a dose of 10^5^ TCID_50_ (Fig. 3J and K). These findings collectively indicate that Lys136 and Lys166 of the E protein jointly contribute to the virulence of JEV. Thus, our results demonstrate that the simultaneous substitution of Lys136 and Lys166 in the JEV E protein attenuates both viral replication and neuroinvasiveness, highlighting the critical role of these residues in JEV pathogenicity.

### K27-linked polyubiquitination of lysine residues K136 and K166 of the JEV E protein in virion enhances viral adsorption to target cells

**Fig 4:**
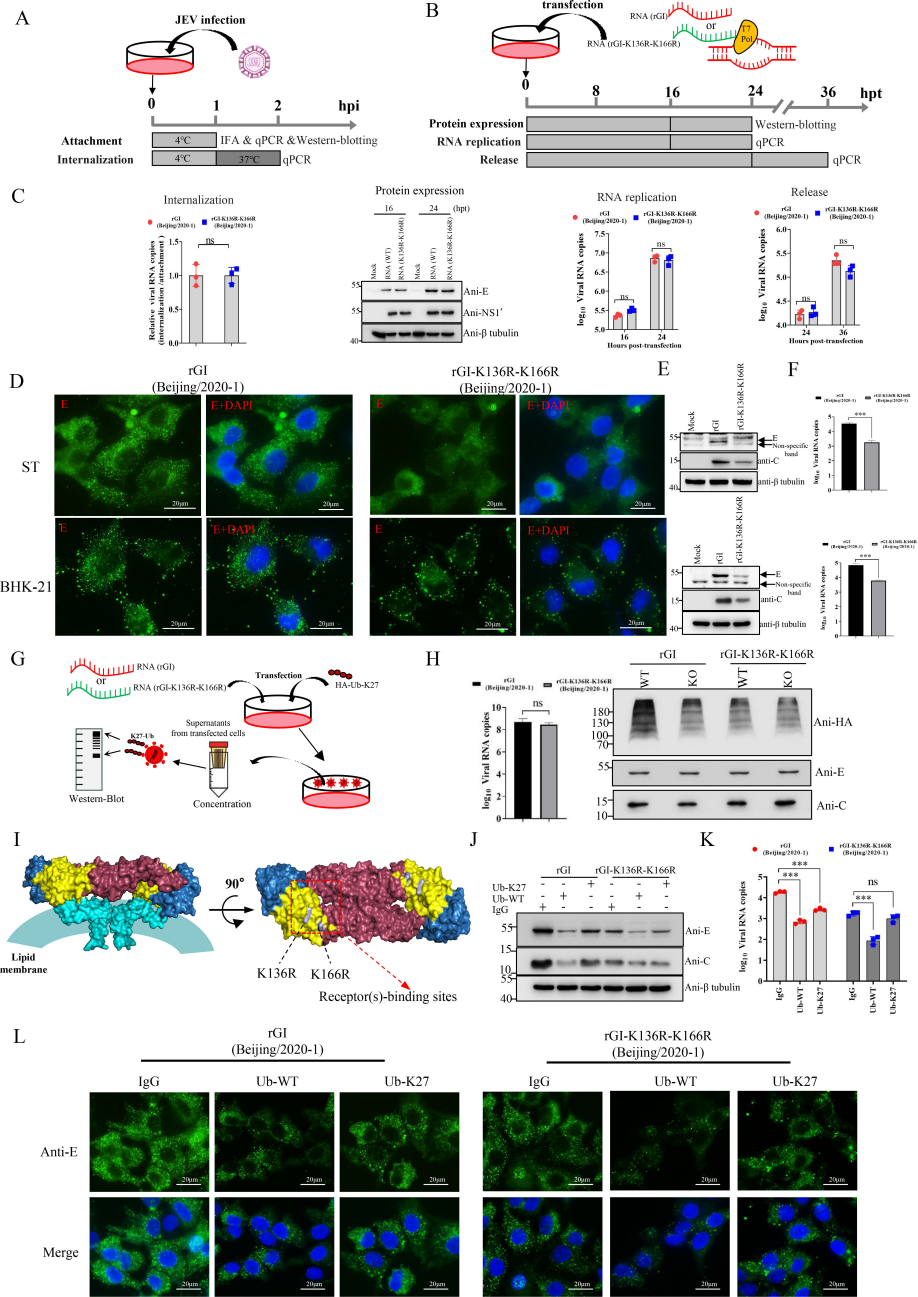
K27-linked polyubiquitination of K136 and K166 in the E protein enhances JEV adsorption to target cells. (**A and B**) Experimental design for examining virus attachment, internalization, protein expression, RNA replication, and release. (**C**) BHK-21 cells were incubated with JEV at 4°C for 1 h to allow adsorption, then shifted to 37°C for 1 h for internalization analysis by qPCR. Equal amounts of JEV genomic RNAs (rGI and rGI-K136R-K166R) were transfected into BHK-21 cells for protein expression, RNA replication, and virion release analysis by qRT-PCR and western blotting. (D, E, and F) To assess viral attachment, BHK-21 or ST cells were incubated with rGI or rGI-K136R-K166R at 4°C for 1 h, followed by washing and analysis by IFA (**D**), western blotting (**E**), and qPCR (**F**). (**G**) Strategy for analyzing K27-linked ubiquitination at K136 and K166 of the E protein in mature JEV particles. (**H**) Purified viruses were lysed in Trizol for viral RNA quantification by qPCR and analyzed for K27-linked polyubiquitination of E-K136 and -K166 by western blot analysis. (**I**) Side view of the averaged E protein heterodimer in mature JEV particles. The red box indicates putative receptor-binding sites. (J, K, and L) rGI and rGI-K136R-K166R were pre-incubated with anti-ubiquitin and anti-ubiquitin (K27-linkage specific) antibodies, or IgG control for 1 h at 37°C, then used to infect BHK-21 cells. After 1 h of incubation at 4 °C, the cells were collected for quantitative analysis by western blotting (**J**), qPCR (**K**), and IFA (L). Data are presented as mean ± SD from three independent experiments. The *P*-value was calculated using the student’s *t*-test. ***, *P* < 0.001; **, *P* < 0.01; *, *P* < 0.05; ns, no significant.

To elucidate the mechanisms by which lys136 and lys166 influence JEV replication, we examined the effect of K136R-K166R double mutation on various stages of the JEV replication cycle, including attachment, internalization, protein expression, RNA replication, and viral release in BHK-21 cells (Fig. 4A and B). Although the K136R-K166R double mutation did not significantly affect internalization, RNA synthesis, protein expression, or viral release (Fig. 4C), it markedly impaired viral attachment. This was evidenced by reduced levels of JEV structural proteins detected by IFA and western blot analysis (Fig. 4D and E), as well as lower viral RNA levels measured by RT-qPCR (Fig. 4F). These findings suggest that K27-linked polyubiquitination at K136 and K166 of the E protein is critical for JEV attachment to permissive cells.

Given that flavivirus particles contain ubiquitinated E protein (26, 36), we further investigated whether the E protein in JEV virions is ubiquitinated at K136 and K166 residues with K27-linked ubiquitin chains. WT (rGI) and mutant (rGI-K136R-K166R) JEV genomic RNAs prepared by *in vitro* transcription were, respectively, transfected into either wild-type or MARCH5-KO BHK-21 cells expressing Ub-K27 (Fig. 4G). At 36 h post-transfection, mature JEV virions released in the culture supernatants were collected and concentrated using an ultrafiltration device (Fig. 4G). The ubiquitination status of the E protein in virions was determined by western blot analysis, and RT-qPCR quantification of viral genomic RNA was conducted to confirm equal input of WT and mutant virions (Fig. 4H). Western blot analysis revealed K27-linked ubiquitination of the E protein in both WT and mutant virions produced in WT and MARCH5-KO cells (Fig. 4H). The level of K27-linked ubiquitination in WT virions was significantly reduced in MARCH5-KO cells, whereas mutant virions exhibited similar ubiquitination levels, regardless of MARCH5 status (Fig. 4H). These results suggest that JEV virions are ubiquitinated with K27-linked polyubiquitin chains and that MARCH5 is essential for the K27-linked ubiquitination of K136 and K166 residues of the E protein.

The entry of flaviviruses into target cells is mediated by the interactions between E protein and cellular receptors (37). The specific motifs on the surface of the JEV E protein determine viral attachment to cellular receptors (9) (Fig. 4I). Structural analysis revealed that K136 and K166 of the E protein are located at the binding interface for the putative receptor(s) (Fig. 4I), and these residues are highly conserved across various JEV strains (Fig. S3; Table S2). To determine whether K27-linked ubiquitination of K136 and K166 directly facilitates viral attachment, we performed an inhibition assay using antibodies against ubiquitin or linkage-specific K27-ubiquitin. Pre-incubation of rGI and rGI-K136R-K166R with anti-ubiquitin antibodies significantly reduced viral attachment as quantified by western blotting, qPCR, and IFA, whereas rabbit IgG control had no effect (Fig. 4J through L). Notably, the inhibitory effect of the anti-K27-ubiquitin antibody was specific to rGI and did not affect rGI-K136R-K166R (Fig. 4J through L), supporting the conclusion that K27-linked ubiquitination of K136 and K166 residues on the E protein enhances JEV attachment to target cells.

### MARCH5 negatively regulates RIG-I-like receptor-mediated IFN-β production

Having established the decisive role of MARCH5 in mediating K27-linked ubiquitination of K136 and K166 on the JEV E protein, we further characterized the growth dynamics of the rGI-K136R-K166R in WT and MARCH5-KO cells across different cell lines. In contrast to the attenuated growth of rGI in MARCH5-KO cells of BHK-21 and ST cells (Fig. 1D and G), the rGI-K136R-K166R exhibited similar viral titers in both WT and MARCH5-KO BHK-21 cells (Fig. 4A and B). However, in ST cells, rGI-K136R-K166R replicated to higher viral titers compared with MARCH5-KO ST cells (Fig.S4C and D). To validate these observations, we generated MARCH5-knockdown cell lines in Vero and PIEC cells. Consistent with the results in ST cells, the knockdown (KD) of MARCH5 in PIEC cells significantly reduced viral titers of rGI-K136R-K166R at 24 hpi and 36 hpi (Fig. S4G and H). In contrast, the knockdown of MARCH5 in Vero cells exhibited no significant effect on the replication of rGI-K136R-K166R (Fig. 4E and F).

**Fig 5:**
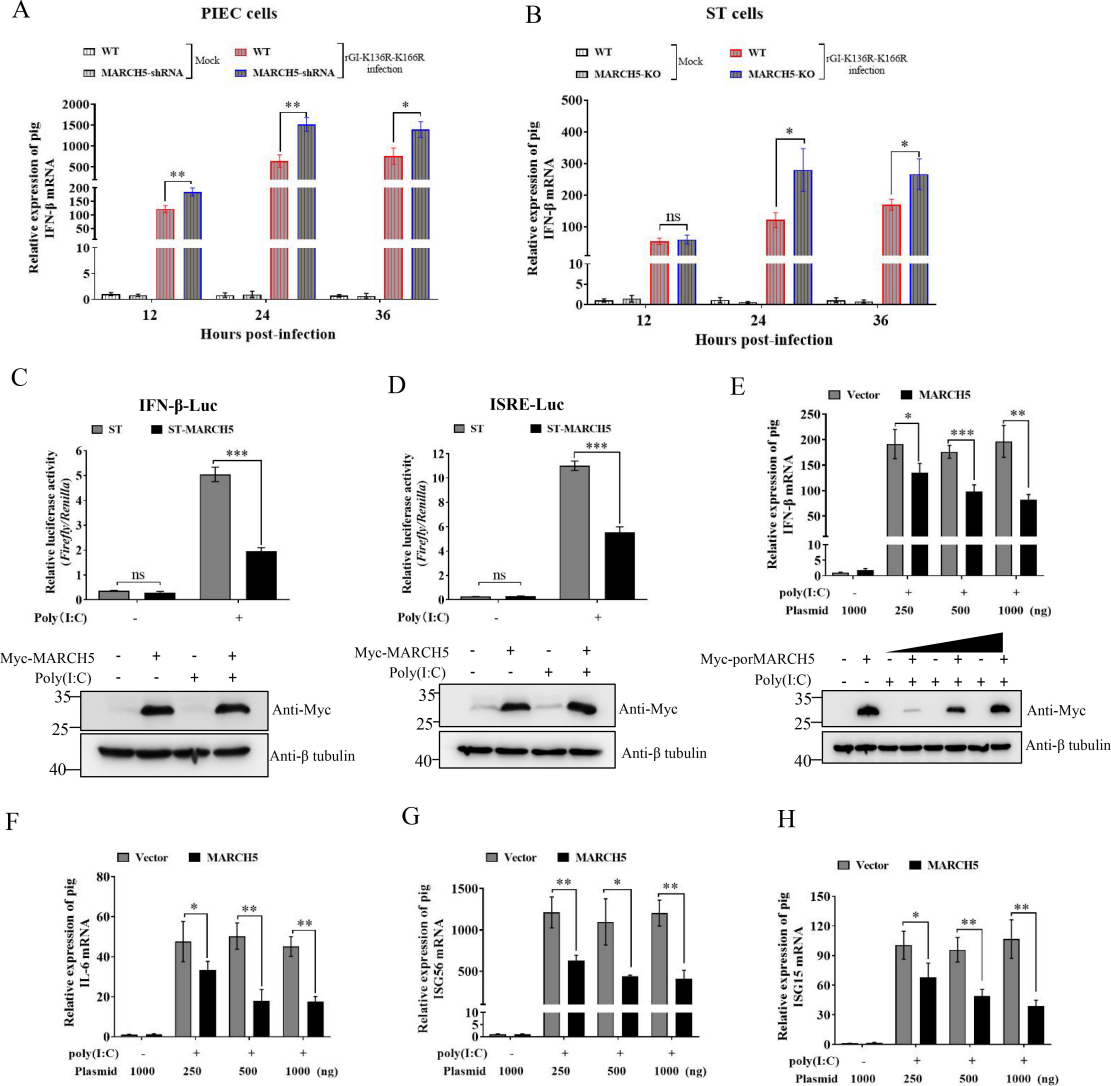
MARCH5 negatively regulates RIG-I-mediated IFN-β signaling. (**A and B**) WT or MARCH5-KO/KD PIEC and ST cells were, respectively, infected with rGI-K136R-K166R at an MOI of 0.01. Cells were harvested at 12, 24, and 36 hpi for IFN-β mRNA quantification by qRT-PCR. (**C**) HEK-293T cells were co-transfected with pIFN-β-luc and pRL-TK, or pISRE-luc and pRL-TK, along with pCMV-MARCH5-Myc, with or without poly(I:C) stimulation. At 24 hpt, IFN-β and ISRE promoter activity was measured using a dual-luciferase reporter assay. (**E–H**), ST cells were transfected with increasing amounts of myc-porMARCH5 (250, 500, and 1,000 ng) and treated with or without poly(I:C) for 24 h. Myc-porMARCH5 expression was detected by western blotting with an anti-Myc antibody. The mRNA levels of IFN-β (**E**), IL6 (**F**), ISG56 (**G**), and ISG15 (**H**) were determined by qRT-PCR. Data are normalized to GAPDH and presented as mean ± SD from three independent experiments. The p-value was calculated using the Student’s *t*-test. ***, *P* < 0.001; **, *P* < 0.01; *, *P* < 0.05; ns, no significant.

Given that type I IFN production is deficient in BHK-21 and Vero cells, we speculated that MARCH5 might suppress type I IFN production in PIEC and ST cells to benefit JEV replication. To test this, we compared IFN-β levels induced by rGI-K136R-K166R infection in WT and MARCH5-KO/KD ST and PIEC cells. The results showed that IFN-β expression was significantly higher in MARCH5-KO ST and MARCH5-KD PIEC cells compared with their WT cells (Fig. 5A and B), suggesting that MARCH5 downregulated IFN-β production in ST and PIEC cells during JEV infection. To confirm these findings, HEK-293T cells were co-transfected with pMyc-MARCH5 and either an ISRE- or IFN-β- luciferase reporter, followed by stimulation with poly(I:C) transfection. Overexpression of MARCH5 markedly inhibited poly(I:C)-triggered activation of the ISRE and IFN-β promoter (Fig. 5C and D), confirming the role of MARCH5 as a negative regulator of type I IFN production.

RIG-I-like receptors are key pathogen recognition receptors that detect viral RNAs and initiate antiviral signaling (38). Since ST and PIEC cells are of porcine origin, we further explored the effect of porcine MARCH5 (porMARCH5) on RLR signaling pathways. As shown in Fig. 5E, porMARCH5 inhibited the production of IFN-β induced by poly(I:C) stimulation at the mRNA levels in a dose-dependent manner. Moreover, the expression of three interferon-stimulated genes (IL-6, ISG56, and ISG15) was downregulated by the overexpression of porMARCH5 in ST cells (Fig. 5F through H). Taken together, these results suggest that MARCH5 negatively regulated IFN-β production in porcine cells, likely by modulating RLR signaling pathways.

### MARCH5 targets MAVS for K48-linked polyubiquitination and degradation

**Fig 6:**
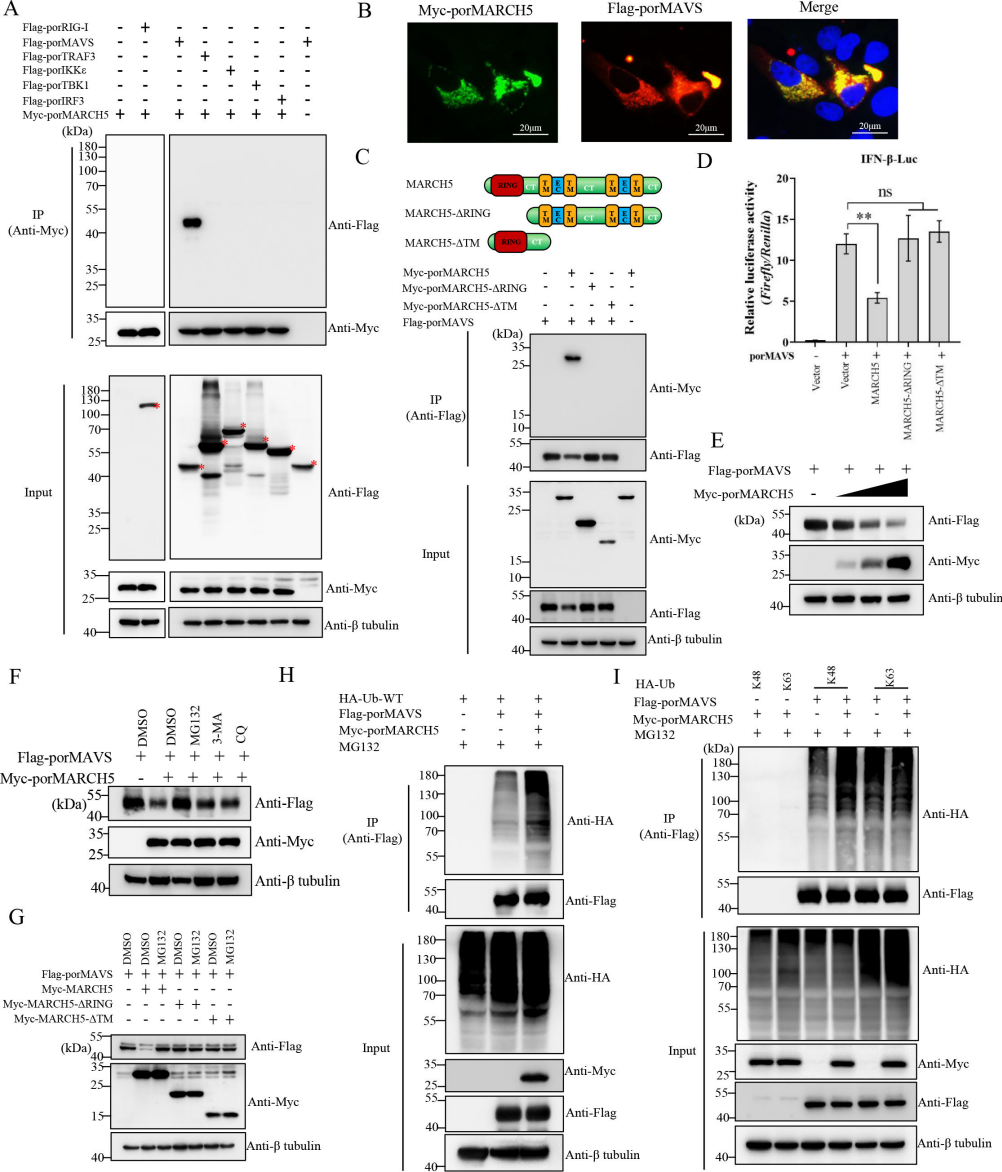
MARCH5 targets MAVS for K48-linked polyubiquitination and degradation. (**A**) HEK-293T were co-transfected with pMyc-porMARCH5 and pFlag-porRIG-I, pFlag-porMAVS, pFlag-porTRAF3, pFlag-porIKKε, pFlag-porTBK1, or pFlag-porIRF3 for 24 h. Cell lysates were collected for co-IP using anti-Myc magnetic beads, and the immunoprecipitated protein complex was analyzed using indicated antibodies by western blotting. (**B**) Confocal microscopy analysis of Myc-porMARCH5 and Flag-porMAVS colocalization in ST cells. (**C**) Co-IP analysis of porMARCH5 or its truncation mutants binding to porMAVS. (**D**) ST cells were co-transfected with pIFN-β-luc, pRL-TK, and pFlag-porMAVS. At 24 hpt, cells were collected to detect IFN-β promoter activity using a dual luciferase reporter assay. Data are presented as mean ± SD from three independent experiments. The *P*-value was calculated using the Student’s *t*-test. **, *P* < 0.01; ns, no significant. (**E**) ST cells were transfected with increasing amounts of Myc-porMARCH5 (250, 500, and 1,000 ng) and pFlag-porMAVS. At 24 hpt, the cells were harvested to detect Myc-porMARCH5 and Flag-porMAVS expression. (**F**) ST cells were co-transfected with plasmids expressing Myc-porMARCH5 and pFlag-porMAVS in the presence or absence of MG132, 3-MA, or CQ. At 24 hpt, cells were harvested to detect Myc-porMARCH5 and Flag-porMAVS expression. (**G**) ST cells were co-transfected with Myc-porMARCH5 or its truncation mutants and Myc-porMAVS, with or without MG132. Flag-porMAVS expression was analyzed by western blotting. (**H and I**) ST cells were co-transfected with pFlag-porMAVS, pHA-Ub-WT, pHA-Ub-K48, or pHA-Ub-K63, along with pMyc-porMARCH5 or empty vector, in the presence of MG132. At 24 hpt, cell lysates were collected for IP using anti-Flag magnetic beads. The ubiquitination of immunoprecipitated porMAVS was analyzed by western blotting using an anti-HA antibody.

Following viral infection, various components of the type I IFN signaling pathway play critical roles in mounting an effective cellular antiviral response. To identify the specific molecule(s) within the porcine RLR signaling pathway regulated by porMARCH5, the interaction between porMARCH5 and key adaptors of the RLR signaling pathway, including porRIG-I, porMAVS, porTRAF3, porIKKε, porTBK1, and porIRF3, was studied using co-IP experiments. The results revealed that porMARCH5 specifically interacted with porMAVS but not with porRIG-I, porTRAF3, porIKKε, porTBK1, and porIRF3 (Fig. 6A). Furthermore, porMARCH5 and porMAVS colocalized in the cytoplasm in ST cells (Fig. 6B). MARCH5 consists of a RING domain and four transmembrane (TM) domains (Fig. 6C). To map the regions of MARCH5 responsible for its interaction with porMAVS, two porMARCH5 mutants with a deletion of the RING domain or TM domains were created. Co-IP assays demonstrated that the absence of either domain of porMARCH5 disrupted the interaction between porMARCH5 and porMAVS (Fig. 6C). Furthermore, neither porMARCH5-ΔRING nor porMARCH5-ΔTM inhibited the activation of the IFN-β promoter induced by porMAVS, unlike the intact porMARCH5 (Fig. 6D), indicating that only full-length porMACH5 can effectively antagonize the RLR signaling pathway.

As E3 ubiquitin ligases, MARCH proteins are often involved in the degradation of target proteins through their enzymatic activity (39, 40). To further elucidate the mechanisms by which porMARCH5 inhibits the RLR signaling pathway, we examined whether porMARCH5 could degrade porMAVS. The expression of porMAVS was downregulated by porMARCH5 in a dose-dependent manner when these proteins were ectopically expressed in ST cells (Fig. 6E). Protein degradation is primarily mediated by the ubiquitin-proteasome pathway or lysosomal pathway (41). To further elucidate the degradation mechanism of porMAVS, we treated cells with the autophagy inhibitors chloroquine (CQ) and 3-methyladenine (3-MA) or the proteasome inhibitor MG132. As shown in Fig. 6F, the porMARCH5-triggered porMAVS degradation was blocked by MG132, but not by CQ and 3-MA, indicating that porMAVS degradation mediated by porMARCH5 occurs via the ubiquitin-proteasome pathway. Furthermore, two porMARCH5 deletion mutants were unable to mediate the degradation of porMAVS (Fig. 6G).

To evaluate whether porMARCH5 ubiquitinates porMAVS via its E3 ligase activity, we determined the ubiquitination status of porMAVS in the presence or absence of porMARCH5 in 293T cells overexpressing HA-tagged ubiquitin (HA-Ub). PorMARCH5 enhanced the ubiquitination of porMAVS in the presence of MG132 (Fig. 6H). Since K48-linked polyubiquitin typically targets proteins for proteasomal degradation (42), we test whether porMARCH5 specifically mediates K48-linked polyubiquitination of porMAVS. The results showed that porMARCH5 markedly increased the K48-linked, but not K63-linked polyubiquitination of porMAVS in the presence of MG132 (Fig. 6I).

In summary, our findings demonstrate that porMARCH5 promotes the K48-linked polyubiquitination of porMAVS, leading to its degradation via the ubiquitin-proteasome pathway.

### MARCH5 ubiquitinates the lysine-286 residue of porMAVS

**Fig 7:**
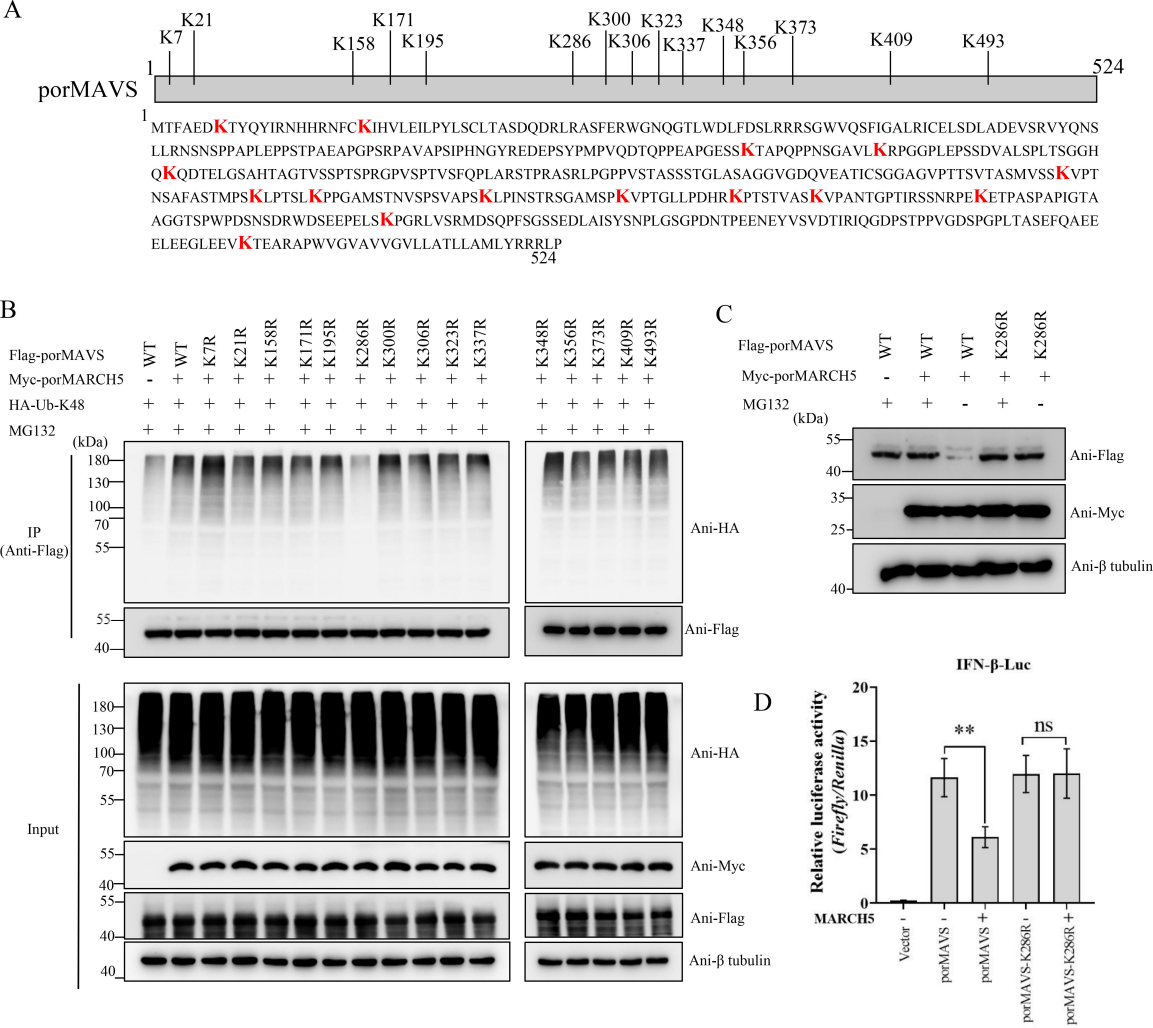
porMARCH5 transfers ubiquitin to Lys286 of porMAVS. (**A**) Schematic representation of potential ubiquitination sites in the E protein. (**B**) ST cells were co-transfected with pFlag-porMAVS or its mutants, pHA-Ub-K48, and pMyc-porMARCH5 in the presence of MG132. At 24 hpi, cell lysates were collected for immunoprecipitation using anti-Flag magnetic beads. The ubiquitination of immunoprecipitated porMAVS was analyzed by western blotting using an anti-HA antibody. (**C**) ST cells were co-transfected with a plasmid expressing Myc-porMARCH5 and pFlag-porMAVS or pFlag-porMAVS-K286R, with or without MG132. At 24 hpt, cells were collected to analyze Myc-porMARCH5 and Flag-porMAVS expression. (**D**) ST cells were co-transfected with pIFN-β-luc, pRL-TK, and pFlag-porMAVS or pFlag-porMAVS-K286R. At 24 hpt, IFN-β promoter activity was measured using a dual-luciferase reporter assay. Data are presented as mean ± SD from three independent experiments. The *P*-value was calculated using the Student’s *t*-test. **P < 0.01; ns, not significant.

Porcine MAVS contains 15 lysine residues (Fig. 7A). To identify the specific lysine residues targeted by porMARCH5 for ubiquitination, we generated a panel of porMAVS mutants with individual lysine mutated to arginine. The ubiquitination level of the porMAVS-K286R mutant was dramatically reduced compared to wild-type porMAVS and other mutants (Fig. 7B), indicating that the K286 residue is the primary ubiquitination site catalyzed by porMARCH5. Additionally, porMARCH5 mediated the degradation of porMAVS but not the porMAVS-K286R mutant (Fig. 7C), confirming the critical role of K286 in this process. We also determined whether porMARCH5 can inhibit the RLR signaling pathway activated by the porMAVS-K286R mutant in ST cells. PorMARCH5, but not its mutant, inhibited the activation of IFN-β promoter stimulated by porMAVS (Fig. 7D). Taken together, our results suggest that porMARCH5 conjugates the K48-linkage polyubiquitin to the K286 residue of porMAVS, which triggers the degradation of porMAVS through the ubiquitin-proteasome pathway, thereby negatively regulating the porcine RLR signaling pathway.

## DISCUSSION

As a subfamily of RING-type E3 ubiquitin ligases, MARCH proteins have been demonstrated to be implicated in many physiological functions in cell surface protein regulation (43, 44), innate immunity signal transduction (18), and autophagy pathways regulation (45). Particularly, MARCH proteins have garnered increasing attention for their dual roles in viral replication, acting as both antiviral and proviral factors through ubiquitination (46, 47). This “double-edged sword” nature is exemplified by their ability to either restrict viral replication by targeting viral envelope proteins for degradation (32, 47) or promote virus replication by suppressing host antiviral immune responses (48). For instance, MARCH2 restricts HIV-1 replication by ubiquitinating viral envelope glycoproteins for degradation (32, 49, 50); however, it also dampens fish type I IFN response by targeting TBK1 for degradation, thereby promoting grass carp reovirus replication (51, 52). MARCH8 exhibits broad antiviral activity against diverse enveloped viruses including influenza A virus (47), SARS-CoV-2 (53), Ebola Virus (54), and pseudorabies virus (55), whereas it is required for the infection of *Flaviviridae* family members, such as hepatitis C virus, DENV, and ZIKV (46). To date, the role of MARCH proteins in JEV infection remains poorly understood. Here, we systematically investigate the functional relevance of MARCH proteins in JEV replication by examining the effects of siRNA-mediated depletion of MARCH proteins on viral infection. We eventually identified MARCH5 as a proviral factor that promotes JEV infection by ubiquitinating the JEV E protein to facilitate efficient viral attachment and by suppressing type I IFN production to promote JEV infection.

MARCH proteins participate in the regulation of viral infection by targeting viral envelope glycoproteins. For viruses such as HIV-1 (33), EBOV (56), and SARS-CoV-2 (53), the reduced expression of viral glycoproteins is a key mechanism of MARCH-mediated antiviral activity. Although often associated with targeting proteins for degradation, ubiquitination mediated by MARCH proteins also promotes intracellular protein-protein interactions. For example, MARCH8-mediated polyubiquitination of HCV NS2 protein is required for the recruitment of ESCRT machinery and subsequent envelopment of HCV (46). In this study, we found that MARCH5 expression is upregulated during JEV infection, and its depletion significantly reduces JEV replication, indicating that MARCH5 plays a proviral role in JEV infection. We demonstrated that MARCH5 specifically interacts with E protein, instead of other structural proteins, and promotes the K27-linked ubiquitination of the E protein. Ubiquitination plays an important role in virus infection via the modification of viral proteins or host defensive factors (57, 58). In flaviviruses, ubiquitination of various proteins, such as C (27), prM (59), E (26, 60), NS1 (61), NS2A (62), NS4A (62), and NS5 (63), has been implicated in viral replication, transmission, and host tropism. Our results reveal that MARCH5 mediates K27-linked ubiquitination of the JEV E protein at lysine residues K136 and K166. Of note, K136 and K166 are not the only ubiquitinated residues, since the double mutation of K136R-K166R reduces but does not eliminate the ubiquitination of E protein. Thus, MARCH5 is partially responsible for the K27-linked ubiquitination of the E protein. We cannot rule out the possibility that MARCH5 targets other lysines for ubiquitination, in addition to lysine residues K136 and K166.

For some flaviviruses, the ubiquitination of viral structural proteins in mature virions is important for virus tropism and pathogenesis (26). In this study, we identified K27-linked polyubiquitination in JEV virions. This ubiquitination of JEV virions was strongly reduced for the rGI-K136R-166R mutant produced in cells with or without MARCH5 expression, as well as in rGI produced in MARCH5-KO cells, suggesting that the K27-linked polyubiquitination of the E protein at residues K136 and K166 is packaged in mature JEV particles. The E protein is one of the most important structural envelope components of flaviviruses due to its essential role in viral binding and entry into host cells (10, 64). Residues K136 and K166 localize at the putative binding interface between the E protein and cellular receptor(s) (9). Simultaneous arginine substitutions at K136 and K166 attenuated the growth of the rGI-K136R-K166R mutant *in vitro*. Consistently, the replication of the rGI-K136R-K166R mutant *in vivo* was also attenuated, as evidenced by lower viral loads in the brains of mice infected with the mutant. An antibody specifically against the K27-linkage polyubiquitin selectively inhibited the attachment of rGI but not rGI-K136R-K166R mutant, suggesting that the ubiquitination at K136 and K166 residues is important for the E protein’s role in viral attachment. Current prevention and control of JEV mainly rely on modified live vaccines (65). Since the virulence of JEV in mice was reduced due to the simultaneous disruption of ubiquitination sites K136 and K166 on the E protein, these sites could serve as targets for mutagenesis to attenuate JEV, providing new insights for the development of live-attenuated vaccines.

Innate immunity is the first-line defense against virus invasion. Accumulating research has evidenced that MARCH proteins play various roles in regulating host antiviral immune responses (19). Regarding its role in innate immunity, MARCH5 has been reported to catalyze the K63-linked polyubiquitination of TANK, which promotes TLR7-mediated NF-κB activation (66). Studies have shown that MARCH5 enhances STING pathway activation by suppressing the polymerization of oxidized STING (67). In this study, we discovered that MARCH5 exerts a suppressive effect on type I IFN production by degrading MAVS through the ubiquitin-proteasome pathway, thereby promoting JEV replication. The discrepancy between our findings and those of previous studies suggests that MARCH5 may exert distinct regulatory effects in different innate immune signaling pathways. Of note, MARCH5 expression was dramatically induced upon JEV infection, suggesting that MARCH5 may form a negative feedback loop with type I IFN during JEV infection. The enhanced expression of MARCH5 induced by JEV infection could further suppress type I IFN production, which in turn facilitates the production of JEV. The covalent conjugation of different types of ubiquitin chains to target proteins has emerged as an important way to regulate protein functions in innate immunity (68). K48-linked polyubiquitination usually marks target proteins for proteasomal degradation, whereas K63-linked polyubiquitination is a non-proteolytic modification that is important for the transduction and function of protein signaling complexes (42). Our data reveal that MARCH5 catalyzes the K48-linked polyubiquitination of MAVS, leading to its proteasomal degradation and subsequent inhibition of the RLR signaling cascades, which are essential for type I IFN production.

**Fig 8:**
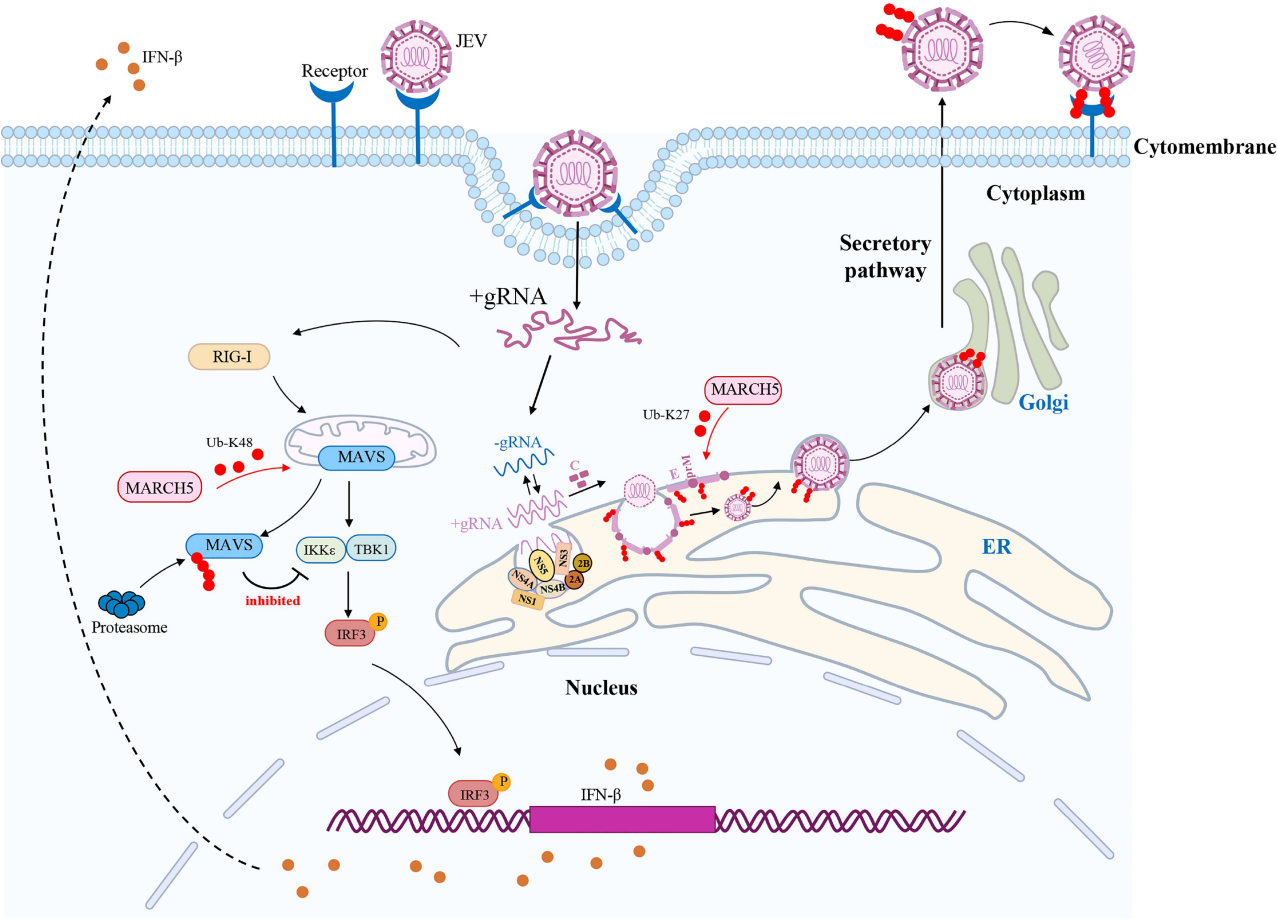
Schematic of MARCH5 regulating JEV replication and blocking IFN production. During JEV infection, MARCH5 interacts with the E protein and catalyzes K27-linked polyubiquitination at residues K136 and K166. Ubiquitinated E in infectious JEV virions enhances viral attachment to host cells, promoting viral replication. MARCH5 also inhibits IFN-β production by inducing K48-linked polyubiquitination and degradation of MAVS.

In conclusion, our study reveals that MARCH5 functions as a proviral factor during JEV infection. As depicted in Fig. 8, MARCH5 promotes JEV infection through two distinct mechanisms, and its expression is further upregulated in response to JEV infection. First, MARCH5 interacts with the viral E protein upon virus infection, mediating K27-linked polyubiquitination at residues K136 and K166, which are located on the ectodomain of the E protein. Moreover, the nucleocapsid core with genomic RNA buds into the ER lumen, forming virus particles with K27-linked polyubiquitinated E protein. These JEV virions are then transported to the Golgi apparatus and released via the conventional secretion pathway. The ubiquitinated E protein in released JEV virions facilitates efficient viral attachment to host cells. Second, MARCH5 suppresses type I IFN production by targeting MAVS for degradation through the ubiquitin-proteasome pathway, thereby promoting JEV infection. Together, these findings highlight the dual role of MARCH5 in enhancing JEV infection and provide new insights into its potential as a therapeutic target.

## MATERIALS AND METHODS

### Cells, virus strains, and antibodies

ST, BHK-21, Vero, PIEC, and HEK-293T cells were obtained from the American Type Culture Collection (ATCC; Manassas, VA, USA) and were cultured in Dulbecco’s modified Eagle’s medium (DMEM; Hyclone, Logan, UT, USA) containing 10% fetal bovine serum (FBS; Sigma, St Louis, MO, USA), 1% penicillin, and streptomycin (Hyclone). The wild-type JEV virulent strain Beijing/2020-1 (GenBank No. OP588746) was propagated in BHK-21 cells maintained in DMEM with 2% FBS and stored in our laboratory (34). The following antibodies were used in this study: mouse anti-JEV NS1' protein, mouse anti-JEV C protein, and rabbit anti-JEV E protein antibodies (GeneTex, Inc., St. Anthony, TX, USA); rabbit anti-MARCH5 protein antibody (Cell Signaling Technology, Danvers, MA, USA); mouse anti-ubiquitin, and the rabbit anti-ubiquitin (linkage-specific K27) antibodies (Abcam, Cambridge MA, USA).

### Virus growth kinetics and viral plaque assay

To assess virus growth kinetics, the cells were seeded in 12-well plates and infected with JEV-WT or JEV-mutants at a multiplicity of infection (MOI) of 0.1 or 0.05. At designated time points after infection, supernatants from infected cells were collected and titrated on BHK-21 cells. Virus titers were calculated as log_10_TCID_50_ (median tissue culture infective dose) per 0.1 mL using the Reed-Muench method.

For viral plaque assay, monolayers of ST and BHK-21 cells in 6-well plates were infected with viruses at a dose of 100–200 TCID_50_. After a 2 h incubation at 37°C, un-adsorbed viruses were removed, and cells were washed three times with 1× PBS. Cells were then overlaid with MEM (Gibco, Grand Island, NY, USA) supplemented with 2% FBS and 1% low-melting-point agarose (Solarbio, Beijing, China). The infected ST cells were cultured for an additional 4 days, fixed with 4% paraformaldehyde for 1 h, and stained with 0.5% crystal violet solution (Solarbio) to visualize and analyze plaque size and morphology.

### Indirect immunofluorescence assay and co-immunoprecipitation assay

The indirect immunofluorescence assay (IFA) was performed following standard protocols. Briefly, ST or BHK-21 cells were seeded in a 24-well plate and grown to 80%–90% confluence. Cells were then co-transfected with plasmids expressing Myc-MARCH5 and Flag-E or Flag-porMAVS. At 24 h post-transfection (hpt), culture supernatants were removed, and the cells were fixed with 4% paraformaldehyde, permeabilized with 0.1% Triton X-100, and blocked with 5% bovine serum albumin (BSA; Solarbio). Cells were incubated with a primary antibody overnight at 4°C, followed by incubation with secondary antibodies for 1 h at 37°C. Nuclei were stained with DAPI (Solarbio), and images were acquired with a confocal epifluorescence microscope (Zeiss LSM900).

For the co-immunoprecipitation assay (Co-IP), HEK-293T cells were transfected with plasmids expressing MARCH5-Myc along with Flag-C, Flag-prM, or Flag-E, or with plasmids expressing porMARCH5-Myc along with Flag-porRIG-I, Flag-porMAVS, Flag-porTRAF3, Flag-porIKKε, Flag-porTBK1, or Flag-porIRF3. At 24 hpt, cells were harvested and lysed with RIPA lysis buffer (Beyotime, Shanghai, China) containing 1 mM phenylmethyl sulfonyl fluoride (PMSF; Beyotime, Shanghai, China). Whole-cell lysates were collected and incubated with anti-Myc or Anti-Flag Protein-A/G magnetic beads (Invitrogen) overnight at 4°C. After three washes, the beads were resuspended in 2× SDS loading buffer and heated at 95°C for 5 min. Protein expression levels were analyzed by western blot analysis.

### CRISPR-Cas9-based genome editing

MARCH5-KO ST and BHK-21 cells were generated using the CRISPR/Cas9 editing method. A guide RNA (gRNA) targeting MARCH5 was designed using the E-CRISP online tool (http://www.e-crisp.org/) (Table S3). Cells with green fluorescence were sorted into 96-well plates by fluorescence-activated cell sorting (FACS) and validated by western blot analysis and genomic DNA sequencing.

### Generation of MARCH5-knockdown cell lines

For MARCH5 silencing, sense and antisense DNA oligonucleotides encoding shRNA (Table S3) targeting MARCH5 mRNA were synthesized, annealed, and inserted into the pLKO.1-Puro vector. Lentivirus expressing shRNAs was produced by co-transfecting HEK-293T cells with pCMV-VSV-G, psPAX2, and the recombinant pLKO.1-MARCH5-shRNA. At 48 hpt, lentivirus-containing supernatants were harvested and filtered through 0.45 µm filters. Vero and PIEC cells were infected with the lentivirus for 48 h, and stable cell lines were selected with puromycin (1.5 µg/mL). Knockdown efficiency was confirmed by western blotting.

### *In vitro* RNA transcription and virus recovery in cells

The JEV infectious cDNA clone pOK-rGI (rGI/Beijing/2020-1) was constructed and stored in our lab (34). Plasmids containing point mutations at E-136 and E-166 were generated with PCR-based site-directed mutagenesis of the pOK-rGI plasmid. For *in vitro* RNA transcription, the JEV cDNA clones were linearized with the restriction endonuclease SalI (NEB, Ipswich, MA, USA). Full-length viral RNA was transcribed *in vitro* from the linearized cDNA template using the mMessage mMachine T7 kit (Invitrogen, Carlsbad, CA, USA). The resulting viral RNA transcripts were transfected into BHK-21 cells in 6-well plates with DMRIE-C reagent (Invitrogen). Supernatants from transfectants showing cytopathic effects (CPE) were collected and amplified in BHK-21 cells. Mutant viruses rescued from BHK cells were plaque-purified and confirmed by Sanger sequencing (GENEWIZ, Suzhou, China).

### Viral adsorption assays

ST or BHK-21 cells were seeded and cultured in glass-bottom dishes overnight to 60%–70% confluence. Cells were washed three times with PBS and infected with viruses at an MOI of 100, followed by incubation for 1 h at 4°C. Unabsorbed viruses were removed by washing five times with PBS. Cells were then collected for western blotting using anti-JEV E and C antibodies, harvested for RT-qPCR analysis to quantify viral RNA copies (5), or fixed with 4% paraformaldehyde and stained with anti-JEV E monoclonal antibody and DAPI.

### Luciferase assays

HEK-293T cells were transfected with pCMV-MARCH5-Myc, along with the IFN-β-Luc reporter and an internal control pRL-TK, for 12 h. Cells were then stimulated with poly(I:C) (1 µg/mL) for 12 h and lysed for luciferase activity measurement. To analyze the effect of MARCH5 on porcine RLR signaling, ST cells were co-transfected with pCMV-porMARCH5-Myc, IFN-β-Luc reporter, pRL-TK, and Flag-tagged porMAVS or its mutant (porMAVS-K286R). At 24 hpt, cell lysates were prepared for luciferase assays. Luciferase activity was measured using the dual-Luc reports assay system (Vazyme, Nanjing, China) on a 96-well microplate luminometer (LumiStation-1800; Flash, Shanghai, China). Data were normalized to transfection efficiency by calculating the ratio between firefly luciferase activity and *Renilla* luciferase activity.

### RNA quantification

Total cellular RNA was extracted with TRIzol reagent (Vazyme) and was reverse-transcribed to cDNA using a cDNA synthesis kit (Vazyme). Quantitative real-time PCR (qPCR) was performed using the SYBR Green PCR Master Mix (Vazyme) on a QuantGene 9600 system (Bioer, Hangzhou, China). Primers used are listed in Table S4. Relative RNA quantities were determined using the ΔΔCt method and normalized to the expression of the GAPDH gene.

### Ubiquitination assay

To analyze the effect of MARCH5 on the ubiquitination of JEV E protein, HEK-293T cells were transfected with plasmids expressing MARCH5-Myc and E-Flag or its mutants, in the presence or absence of HA-tagged ubiquitin or its mutants. Whole-cell lysates were immunoprecipitated with anti-Flag magnetic beads and analyzed by western blot analysis using anti-HA and anti-Flag antibodies. To analyze the effect of MARCH5 on the K27-linked ubiquitination of JEV particles, WT or MARCH5-KO BHK-21 cells were co-transfected with pRK5-HA-Ub-K27 and genomic RNA of JEV-WT or its mutant (rGI-K136R-K166R). At 60 hpt, supernatants were harvested, and JEV particles were concentrated and purified by sucrose gradient density centrifugation. Purified particles were analyzed by western blot analysis with anti-HA or anti-E antibodies.

To evaluate the effect of porMARCH5 on porMAVS ubiquitination, HEK-293T cells were transfected with plasmids expressing porMARCH5-Myc and porMAVS-Flag or its mutants, in the presence or absence of HA-tagged ubiquitin or its mutants. Whole-cell lysates were immunoprecipitated with the anti-Flag magnetic beads and analyzed by western blot analysis with anti-HA or anti-Flag antibodies.

### Animal experiments

Three-week-old C57BL/6 mice were purchased from the Laboratory Animal Center of Yangzhou University and randomly divided into four groups (5 mice per group). Mice were intraperitoneally injected with rGI, rGI-K136R, rGI-K166R, or rGI-K136R-K166R at doses of 10^3^ or 10^5^ TCID_50_ per animal, or with equal volumes of DMEM as a control. Mice were monitored daily for mortality and clinical signs of disease.

### Homology modeling

The JEV E protein structure was modeled with SWISS-MODEL (https://swissmodel.expasy.org/). The E structure (Protein Data Bank accession number 5MV1) was visualized and analyzed using PyMOL (http://www.pymol.org) and SPDBV (DeepView) software (https://spdbv.unil.ch/).

### Statistical analyses

Statistical analyses were performed using GraphPad Prism software (version 8.0.1, San Diego, CA, USA). Quantitative data were represented as means ± standard deviations (SD) from at least three independent experiments. A *P*-value < 0.05 was considered statistically significant.

## SUPPLEMENTAL MATERIAL

Fig. S1 mbio.00208-25-s0001.tif The efficiency of siRNA knockdown and proliferation of WT and MARCH5-KO BHK-21 and ST cell lines.

Fig. S2 mbio.00208-25-s0002.tif Relative expression of MARCH5 during JEV infection.

Fig. S3 mbio.00208-25-s0003.tif Conservation analysis of K136 and K166 amino acid sites of the E protein across various strains.

Fig. S4 mbio.00208-25-s0004.tif Replication ability of rGI-K136R-K166R in WT or MARCH5-KO BHK-21 and ST cells and in WT or MARCH5-shRNA Vero and PIEC cells.

Table S1 mbio.00208-25-s0005.docx Sequences of siRNAs used in the study.

Table S2 mbio.00208-25-s0006.docx Information of JEV strains used for multiple sequence alignment.

Table S3 mbio.00208-25-s0007.docx The sequences of gRNA and shRNA used in this study.

Table S4 mbio.00208-25-s0008.docx The primers used for PCR and qPCR in this study.

ASM does not own the copyrights to Supplemental Material that may be linked to, or accessed through, an article. The authors have granted ASM a non-exclusive, world-wide license to publish the Supplemental Material files. Please contact the corresponding author directly for reuse.
