# Supplementary figures and images for: E3 ubiquitin ligase MARCH5 positively regulates Japanese encephalitis virus infection by catalyzing the K27-linked polyubiquitination of viral E protein and inhibiting MAVS-mediated type I interferon production
Source: mBio. 2025 Mar 12;16(4):e00208-25. doi: 10.1128/mbio.00208-25 (PMC11980370; doi:10.1128/mbio.00208-25)

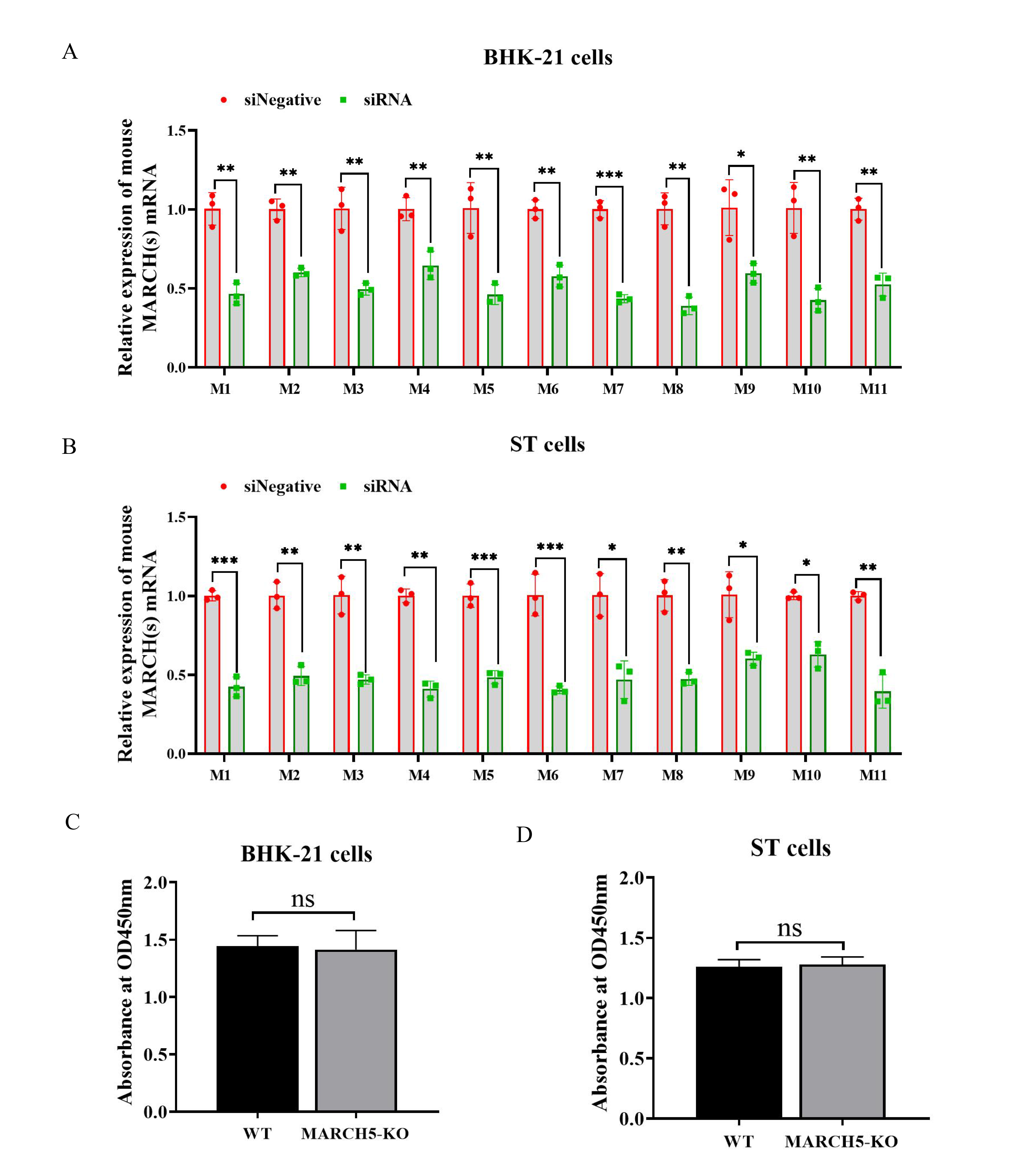

Supplement: Fig. S1 — The efficiency of siRNA knockdown and proliferation of WT and MARCH5-KO BHK-21 and ST cell lines. [file mbio.00208-25-s0001.tif]

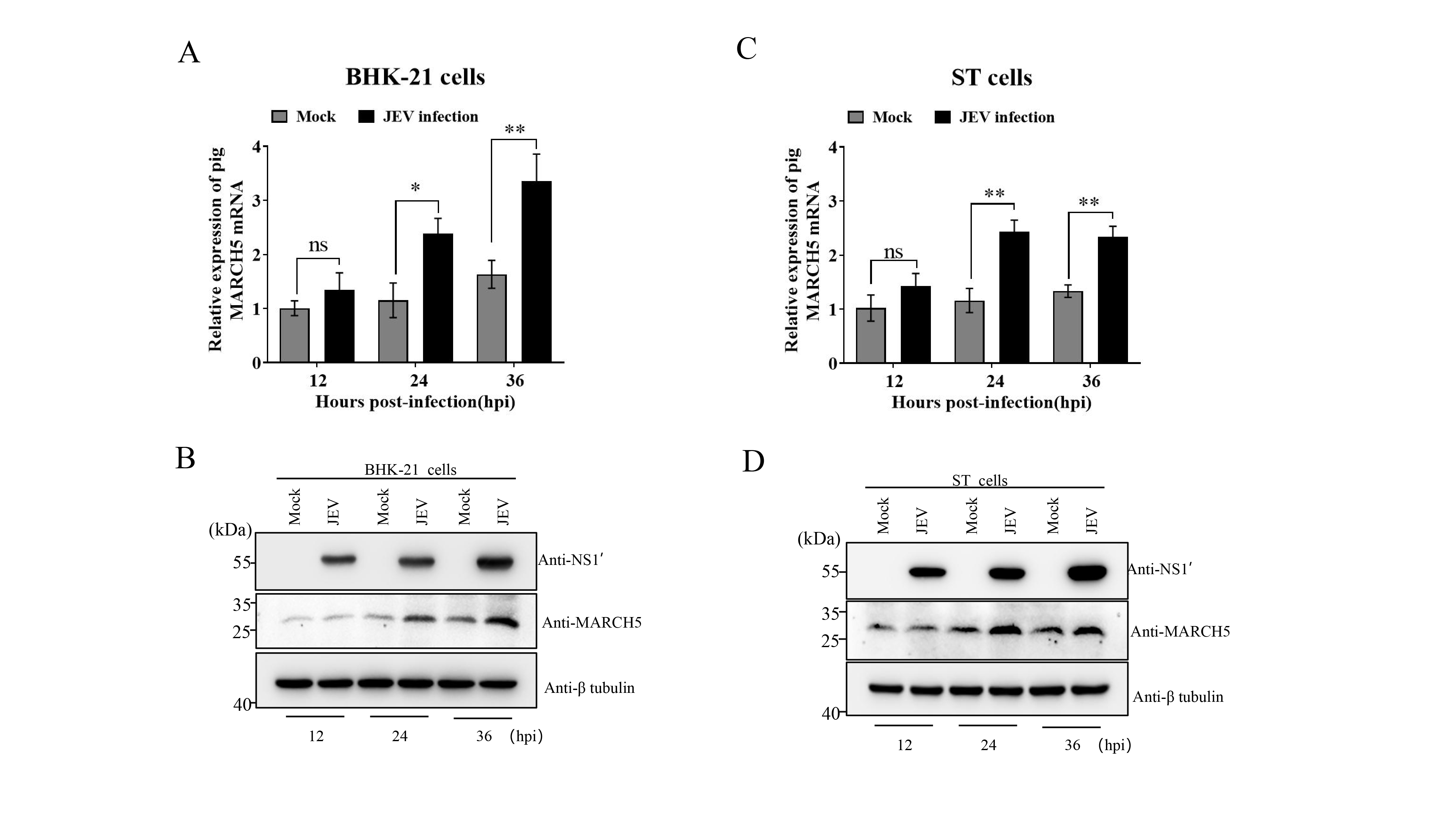

Supplement: Fig. S2 — Relative expression of MARCH5 during JEV infection. [file mbio.00208-25-s0002.tif]

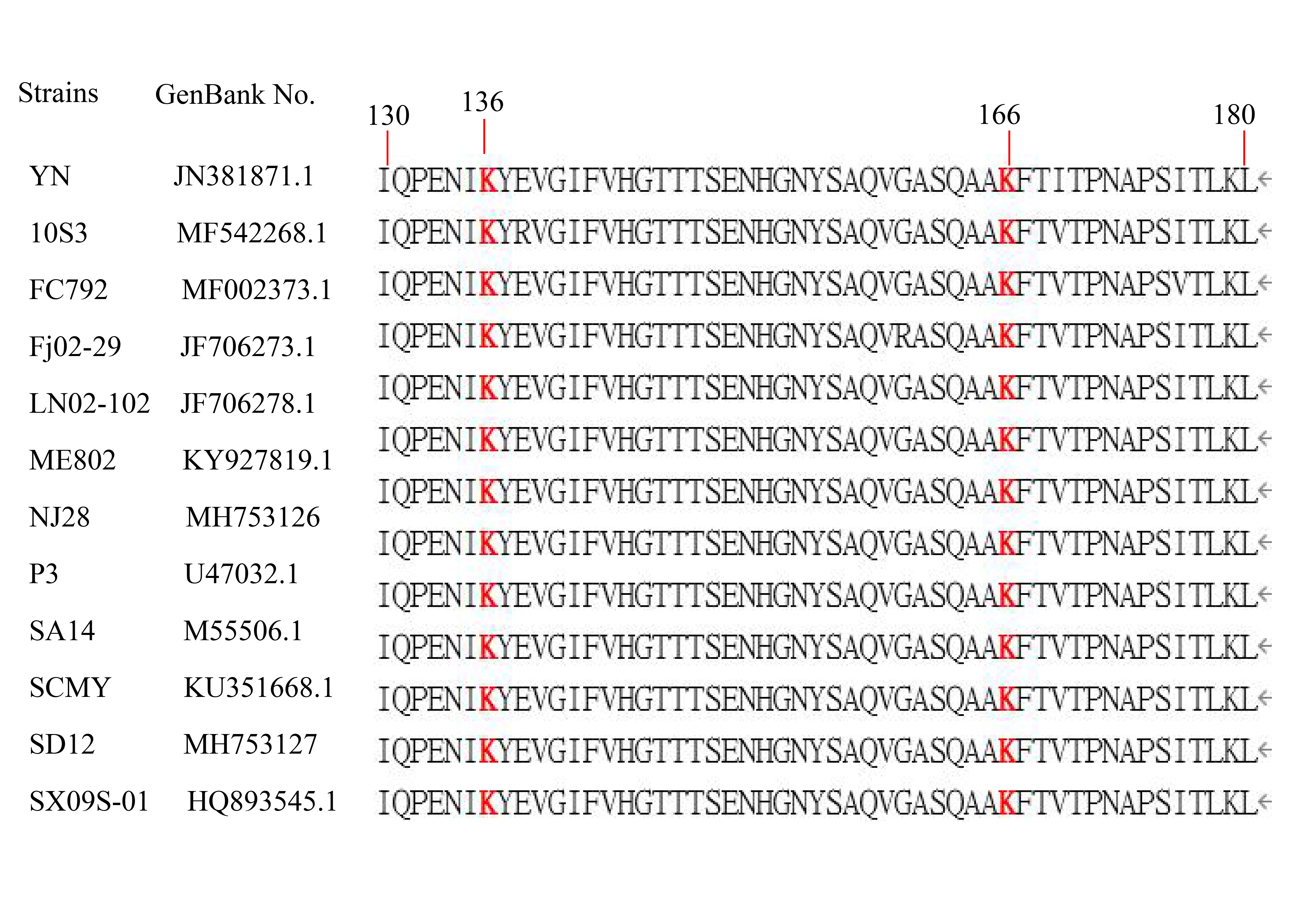

Supplement: Fig. S3 — Conservation analysis of K136 and K166 amino acid sites of the E protein across various strains. [file mbio.00208-25-s0003.tif]

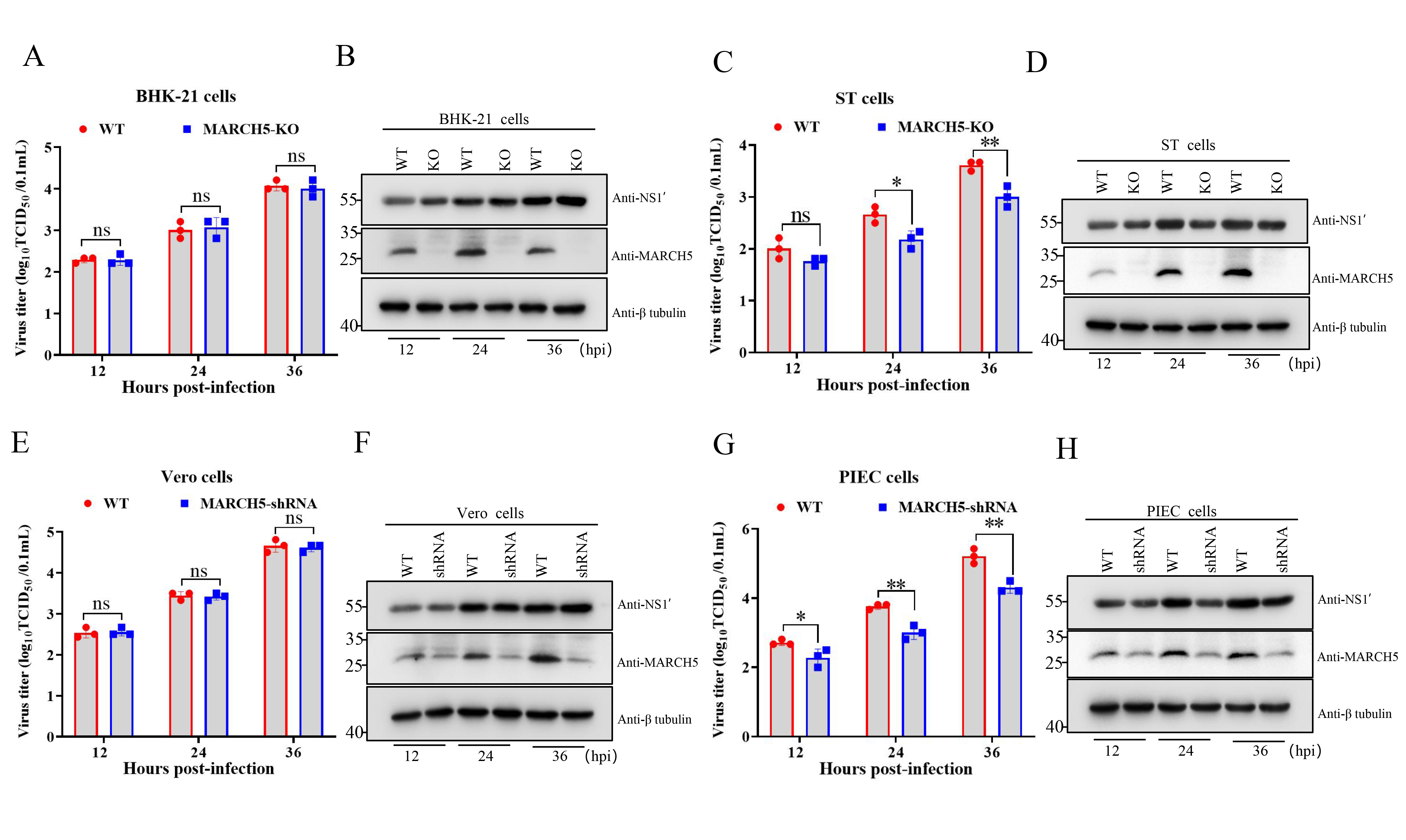

Supplement: Fig. S4 — Replication ability of rGI-K136R-K166R in WT or MARCH5-KO BHK-21 and ST cells and in WT or MARCH5-shRNA Vero and PIEC cells. [file mbio.00208-25-s0004.tif]
