# Supplementary material for: E3 ubiquitin ligase MARCH5 positively regulates Japanese encephalitis virus infection by catalyzing the K27-linked polyubiquitination of viral E protein and inhibiting MAVS-mediated type I interferon production
Source: mBio. 2025 Mar 12;16(4):e00208-25. doi: 10.1128/mbio.00208-25 (PMC11980370; doi:10.1128/mbio.00208-25)
Supplement: Table S1 — Sequences of siRNAs used in the study. [file mbio.00208-25-s0005.docx]

**S1 Table. Sequences of siRNAs used in the study.**

| siRNAs | Sequence (5′ → 3′) | |
| --- | --- | --- |
|  | Pig | Mouse |
| siMARCH1 | GACAUCUGCAGAUCCUCACAUGTT | CAGUCAAGGUUAUCUGUCUGCCTT |
|  | CAUGUGAGGAUCUGCAGAUGUCTT | GGCAGACAGAUAACCUUGACUGTT |
| siMARCH2 | CUGUGUGACUGCUCUGACAGCTT | GUAUGUGGCACAGGUGACCUCTT |
|  | GCUGUCAGAGCAGUCACACAGTT | GAGGUCACCUGUGCCACAUACTT |
| siMARCH3 | GUCAUGCAAGUGUCAGCCAAGGTT | UCAAUGACCGGCCGAUGUGCAGTT |
|  | CCUUGGCUGACACUUGCAUGACTT | CUGCACAUCGGCCGGUCAUUGATT |
| siMARCH4 | CAGAUGUUGCGCCACCAGGGUCTT | CUAUUGCUGUGGAUUGUGCACTT |
|  | GACCCUGGUGGCGCAACAUCUGTT | GUGCACAAUCCACAGCAAUAGTT |
| siMARCH5 | GCCACUGAUGAAGAUGAUAGATT | ACAGCACAGCCAGAGUGGCCUGTT |
|  | UCUAUCAUCUUCAUCAGUGGCTT | CAGGCCACUCUGGCUGUGCUGUTT |
| siMARCH6 | GUAUGUACUGGCAGUAUUAAGUTT | GCUGGACUGGUUACAAGUAUUGTT |
|  | ACUUAAUACUGCCAGUACAUACTT | CAAUACUUGUAACCAGUCCAGCTT |
| siMARCH7 | UAUAGUGAGUCUGAGAUAACUCTT | GUCUGCAUCAGCAUCUGCAUCATT |
|  | GAGUUAUCUCAGACUCACUAUATT | UGAUGCAGAUGCUGAUGCAGACTT |
| siMARCH8 | UCCAUGAGUCAUUCAAGUAACATT | CUAGAGGCUACAGAAGCAAGACTT |
|  | UGUUACUUGAAUGACUCAUGGATT | GUCUUGCUUCUGUAGCCUCUAGTT |
| siMARCH9 | CUAUCGCUCAGCAGCAGCCUGTT | CAUUGCAGUGGCAGGCCAUCUTT |
|  | CAGGCUGCUGCUGAGCGAUAGTT | AGAUGGCCUGCCACUGCAAUGTT |
| siMARCH10 | UGGCCGAACCAAGAUUAUCUGCTT | CCUCAGCAGCACAGGCACCAATT |
|  | GCAGAUAAUCUUGGUUCGGCCATT | UUGGUGCCUGUGCUGCUGAGGTT |
| siMARCH11 | ACGAGCAGCCAGCGGAGGUGGUTT | AGAUGAUUGCUGUAAUCCUAGGTT |
|  | ACCACCUCCGCUGGCUGCUCGUTT | CCUAGGAUUACAGCAAUCAUCUTT |
| siNegative | CUUAAUCCACUACCGAAUGAGATT | |
|  | UCUCAUUCGGUAGUGGAUUAAGTT | |
