# Supplementary material for: E3 ubiquitin ligase MARCH5 positively regulates Japanese encephalitis virus infection by catalyzing the K27-linked polyubiquitination of viral E protein and inhibiting MAVS-mediated type I interferon production
Source: mBio. 2025 Mar 12;16(4):e00208-25. doi: 10.1128/mbio.00208-25 (PMC11980370; doi:10.1128/mbio.00208-25)
Supplement: Table S2 — Information of JEV strains used for multiple sequence alignment. [file mbio.00208-25-s0006.docx]

**S2 Table. Information of JEV strains used for multiple sequence alignment**

| Strains | GenBank No. | Source | Date | Country/region |
| --- | --- | --- | --- | --- |
| SD12 | MH753127 | Pig | 2015 | China |
| 10S3 | MF542268.1 | Pig | 2013 | China |
| LN02-102 | JF706278.1 | *Culex modestus* | 2002 | China |
| ME802 | KY927819.1 | *Homo sapiens* | 2013 | Cambodia |
| SCMY | KU351668.1 | Pig | 2014 | China |
| SX09S-01 | HQ893545.1 | Pig | 2009 | China |
| N28 | MH753126 | Pig | 2015 | China |
| YN | JN381871.1 | *Homo sapiens* | 2011 | China |
| SA-14 | M55506.1 | Mosquito | 1954 | China |
| Fj02-29 | JF706273.1 | Pig | 2002 | China |
| P3 | U47032.1 | *Homo sapiens* | 1949 | China |
| FC792 | MF002373.1 | Pig | 2016 | China |
