# Supplementary material for: E3 ubiquitin ligase MARCH5 positively regulates Japanese encephalitis virus infection by catalyzing the K27-linked polyubiquitination of viral E protein and inhibiting MAVS-mediated type I interferon production
Source: mBio. 2025 Mar 12;16(4):e00208-25. doi: 10.1128/mbio.00208-25 (PMC11980370; doi:10.1128/mbio.00208-25)
Supplement: Table S3 — The sequences of gRNA and shRNA used in this study. [file mbio.00208-25-s0007.docx]

|  | Sequences (5'-3')  **S3 Table. The sequences of gRNA and shRNA used in this study.** |
| --- | --- |
| Pig MARCH5 gRNA | AGCCCTACAGCAGATGCTGG |
| Mouse MARCH5 gRNA | GCATCTGTTGAAGGGCTTGG |
| Pig MARCH5-shRNA | CTGATATTAGGCAAGATGATTCGTCAAGAGCGAATCATCTTGCCTAATATCAGTTTTTT |
| Monkey MARCH5-shRNA | GACAGCTGTGACTTATGGAGCAGTCAAGAGCTGCTCCATAAGTCACAGCTGTCTTTTTTT |
