# Supplementary material for: E3 ubiquitin ligase MARCH5 positively regulates Japanese encephalitis virus infection by catalyzing the K27-linked polyubiquitination of viral E protein and inhibiting MAVS-mediated type I interferon production
Source: mBio. 2025 Mar 12;16(4):e00208-25. doi: 10.1128/mbio.00208-25 (PMC11980370; doi:10.1128/mbio.00208-25)
Supplement: Table S4 — The primers used for PCR and qPCR in this study. [file mbio.00208-25-s0008.docx]

| Plasmids  **S4 Table. The primers used for PCR and qPCR in this study.** | PCR Primers (5'-3') |
| --- | --- |
| pOK-rGI-K136R / pOK-rGI-K136R-K166R | F: CAGAGAACATCAGGTACGAGGTTGG |
|  | R: CAACCTCGTACCTGATGTTCTCTGGT |
| pOK-rGI-K166R / pOK-rGI-K136R-K166R | F: TCTCAAGCAGCAAGGTTTACTGTAA |
|  | R: TACAGTAAACCTTGCTGCTTGAGAC |
| pFlag-E-ΔEDI | F1:CAACTTGCTGAAGTCAGGAGTTACTGCTA |
|  | R1:TCAGTCCACTCCTCTCTGGTTGGATCATTCTTCCA |
|  | F2:ATCCAACCAGAGAGGAGTGGACTGAACACTGAAG |
|  | R2:CATCTCAACCAGTGTCAATTTCACTGAGCTCGAGTA |
|  | F3:AGTGAAATTGACACTGGTTGAGATGGAACCTCCCT |
|  | R3:TTAGGCATGCACATTGGTCGCTAAAAAC |
| pFlag-E-ΔEDII | F1:TTTAACTGTCTGGGAATGGGGAAT |
|  | R1:TGTACTTGATGTTGCTAGCTTCAATGTTGATCATG |
|  | F2:TTGAAGCTAGCAACATCAAGTACAGGGTTGGC |
|  | R2:AGGTGACCTGATGGTTCACAATCCAGTGTGAC |
|  | F3:GATTGTGAACCATCAGGTCACCTGAAATGCAGGC |
| pFlag-E-ΔEDIII | R:TTACACCTTTGAGTTGGAGCTAGATGTT |
| pMyc-MARCH5-ΔRING | F:TCGGTCGACCAATGCTGAATACTTAATAGTTTTTCC |
|  | R:CCGCGGCCGCCTATGCTTCTTCTTGCTCTGGATAATTTAGAATTTT |
| pMyc-MARCH5-ΔTM | F:TCGGTCGACCAATGCTGAATACTTAATAGTTTTTCC |
| pFlag-E-K38R | F:TTATGGCAAACGACCGACCAACACTAG |
|  | R:CTAGTGTTGGTCGGTCGTTTGCCATAA |
| pFlag-E-K136R | F:CCAGAGAACATCCGGTACAGGGTTG |
|  | R:CAACCCTGTACCGGATGTTCTCTGG |
| pFlag-E-K166R | F:GTCTCAAGCAGCACGGTTTACTGTAACTCCAA |
|  | R:TTGGAGTTACAGTAAACCGTGCTGCTTGAGAC |
| pFlag-E-K179R | F:TTCAATAACCCTCCGGCTTGGTGATTAT |
|  | R:ATAATCACCAAGCCGGAGGGTTATTGAA |
| pFlag-E-K286R | F:TCAGGTCACCTGCGATGCAGGCTAAAA |
|  | R:TTTTAGCCTGCATCGCAGGTGACCTGA |
| pFlag-E-K290R | F:TAAAAATGGACCGACTGGCTCTGAAGGG |
|  | R:CCCTTCAGAGCCAGTCGGTCCATTTTTA |
| pFlag-E-K293R | F:ACTGGCTCTGCGGGGCACGACTTA |
|  | R:TAAGTCGTGCCCCGCAGAGCCAGT |
| pFlag-E-K297R | F:ATGTGTACAGAACGATTCTCGTTCGC |
|  | R:GCGAACGAGAATCGTTCTGTACACAT |
| pFlag-porMAVS-K7R | F:TTTGCCGAGGACCGGACTTATCAGT |
|  | R:ACTGATAAGTCCGGTCCTCGGCAAA |
| pFlag-porMAVS-K21R | F:GAATTTTTGCCGGATCCATGTTC |
|  | R:GAACATGGATCCGGCAAAAATTC |
| pFlag-porMAVS-K158R | F:GGAGAGAGTTCACGGACAGCGCCACAGCC |
|  | R:GGCTGTGGCGCTGTCCGTGAACTCTCTCC |
| pFlag-porMAVS-K171R | F:GGGCTGTCCTGCGGAGGCCAGGT |
|  | R:ACCTGGCCTCCGCAGGACAGCCC |
| pFlag-porMAVS-K195R | F:GTGGGCATCAGCGGCAGGACACAGAACT |
|  | R:AGTTCTGTGTCCTGCCGCTGATGCCCAC |
| pFlag-porMAVS-K286R | F:ATGGTGTCCTCTCGAGTGCCCACCAACTC |
|  | R:GAGTTGGTGGGCACTCGAGAGGACACCAT |
| pFlag-porMAVS-K300R | F:CACCATGCCTTCCCGGTTGCCCACCAGC |
|  | R:GCTGGTGGGCAACCGGGAAGGCATGGTG |
| pFlag-porMAVS-K306R | F:CACCAGCCTCCGGCCCCCTGGTGCA |
|  | R:TGCACCAGGGGGCCGGAGGCTGGTG |
| pFlag-porMAVS-K323R | F:GTGTAGCACCATCCCGATTGCCCATCAACTC |
|  | R:GAGTTGATGGGCAATCGGGATGGTGCTACAC |
| pFlag-porMAVS -K337R | F:GCAATGTCACCCCGAGTGCCTACTGGC |
|  | R:GCCAGTAGGCACTCGGGGTGACATTGC |
| pFlag-porMAVS-K348R | F:TGCCGGACCACAGGCGACCTACAAGCAC |
|  | R:GTGCTTGTAGGTCGCCTGTGGTCCGGCA |
| pFlag-porMAVS-K356R | F:CGGTGGCCAGCCGGGTGCCTGCCAAC |
|  | R:GTTGGCAGGCACCCGGCTGGCCACCG |
| pFlag-porMAVS-K373R | F:GCAGCAACCGTCCTGAACGGGAGACTCCAGCATC |
|  | R:GATGCTGGAGTCTCCCGTTCAGGACGGTTGCTGC |
| pFlag-porMAVS-K409R | F:GCCAGAGCTGAGCCGGCCTGGTAGGCTGGT |
|  | R:ACCAGCCTACCAGGCCGGCTCAGCTCTGGC |
| pFlag-porMAVS-K493R | F:TGGAAGAGGTCCGGACTGAGGCTAGGG |
|  | R:CCCTAGCCTCAGTCCGGACCTCTTCCA |
| Gene name | qPCR primers (5'-3') |
| JEV | F:GGGTCAGATCCGTCACTAGAC |
|  | R:ACGACGAACGTGGAGTTGGC |
| Pig MARCH5 | F:TTTTGCTGCAGCGGGAATAAT |
|  | R:GCGCCATAGTCTAAGCACATAATC |
| Mouse MARCH5 | F:TATGTCTTGGATCTTGCAGA |
|  | R:TCAGCTCGCTCCATGACATCT |
| Pig GAPDH | F:CAATGGAAAGGCCATCACCA |
|  | R:CCGGCCTTCTCCATGGTCGT |
| Mouse GAPDH | F: GATGCTGGCGCCGAGTATGT |
|  | R:GTCTTGGTTCACACCCATC |
| Pig IFN-β | F:ATGATGTGCTTCGATACCAA |
|  | R:TTGTGGTGGTTGCATAATCT |
| Pig IL-6 | F:CGCCTGGAAGAAGATGCCAA |
|  | R:TTCTCATACTTCTCACACAT |
| Pig ISG56 | F:GGCCTATGTGAAACACCTGA |
|  | R:ATAGTTGCCCCAGGTAACCA |
| Pig ISG15 | F:ATCGGACCTGAAGCAGCAGA |
|  | R:TGCACCATCAACAGGACTGT |
